# Nonstructural proteins nsp2TF and nsp2N of porcine reproductive and respiratory syndrome virus (PRRSV) play important roles in suppressing host innate immune responses

**DOI:** 10.1016/j.virol.2017.12.017

**Published:** 2018-04

**Authors:** Y. Li, P. Shang, D. Shyu, C. Carrillo, P. Naraghi-Arani, Crystal J. Jaing, G.J. Renukaradhya, A.E. Firth, E.J. Snijder, Y. Fang

**Affiliations:** a Department of Diagnostic Medicine and Pathobiology, Kansas State Veterinary Diagnostic Laboratory, College of Veterinary Medicine, Kansas State University, Manhattan, KS 66506, USA; b Food Animal Health Research Program (FAHRP), Veterinary Preventive Medicine, The Ohio State University, Wooster, OH 44691, USA; c Physical & Life Sciences Directorate, Lawrence Livermore National Laboratory, Livermore, CA 94550, USA; d Department of Pathology, University of Cambridge, Cambridge CB2 1QP, United Kingdom; e Molecular Virology Laboratory, Department of Medical Microbiology, Leiden University Medical Center, 2333 ZA Leiden, The Netherlands

**Keywords:** PRRSV, nsp2TF, nsp2N, Ribosomal frameshift, Innate immune response

## Abstract

Recently, we identified a unique -2/-1 ribosomal frameshift mechanism in PRRSV, which yields two truncated forms of nonstructural protein (nsp) 2 variants, nsp2TF and nsp2N. Here, *in vitro* expression of individual PRRSV nsp2TF and nsp2N demonstrated their ability to suppress cellular innate immune responses in transfected cells. Two recombinant viruses were further analyzed, in which either nsp2TF was C-terminally truncated (vKO1) or expression of both nsp2TF and nsp2N was knocked out (vKO2). Host cellular mRNA profiling showed that a panel of cellular immune genes, in particular those involved in innate immunity, was upregulated in cells infected with vKO1 and vKO2. Compared to the wild-type virus, vKO1 and vKO2 expedited the IFN-α response and increased NK cell cytotoxicity, and subsequently enhanced T cell immune responses in infected pigs. Our data strongly implicate nsp2TF/nsp2N in arteriviral immune evasion and demonstrate that nsp2TF/nsp2N-deficient PRRSV is less capable of counteracting host innate immune responses.

## Highlights

• *In vitro* expression of PRRSV nsp2TF/nsp2N affects cellular innate immune response.
• Nsp2TF/nsp2N suppressed immune genes expression in PRRSV-infected cells.
• Nsp2TF/nsp2N-deficient mutants showed attenuated growth *in vivo.*
• Nsp2TF/nsp2N-deficient mutants induced augmented immune responses in infected pigs.

## 1 Introduction

The innate immune response provides the first line of defense against intruding pathogens. It is essential for the initial control of infection and allows time for launching an adequate adaptive immune response. The type I interferon (IFN) system is a key component of the innate immune response (Haller et al., 2006, Randall and Goodbourn, 2008). Initially, pathogen-associated molecular patterns, like double-stranded RNA (dsRNA) in the case of RNA virus infection, are recognized by host cell receptors to activate protein signaling cascades, which results in the activation of transcription factors, including IRF3, NF-κB, and ATF-2/c JUN. Their coordinated activation leads to the formation of transcriptionally competent enhanceosomes in the cell nucleus, which induce the expression of type I IFNs. After being secreted from infected cells, type I IFNs bind to receptors on the surface of adjacent cells to activate the so-called JAK-STAT signaling pathway. This induces the transcription of a range of interferon-stimulated genes (ISGs), whose products function as effector molecules in the host cell response to viral infection (Raftery and Stevenson, 2017, Schoggins and Rice, 2011).

To counteract the host cell's defense mechanisms, many viruses express proteins that suppress or delay innate immune responses (Haller et al., 2006, Randall and Goodbourn, 2008, Versteeg and Garcia-Sastre, 2010). Previous studies have implicated multiple proteins of porcine reproductive and respiratory syndrome virus (PRRSV) in suppressing the type I IFN response [reviewed in (Fang and Snijder, 2010, Ke and Yoo, 2017, Lunney et al., 2016, Snijder et al., 2013)]. PRRSV is an enveloped, positive-stranded RNA virus, which belongs to the order *Nidovirales,* family *Arteriviridae*. The family also includes equine arteritis virus (EAV), mouse lactate dehydrogenase-elevating virus (LDV), simian hemorrhagic fever virus (SHFV) [reviewed in (Snijder et al., 2013)], and a number of more recently identified members, many of which are of simian origin (Gulyaeva et al., 2017, Kuhn et al., 2016). Historically, PRRSV isolates have been divided into two distinct genotypes, European genotype (Type 1) and North American genotype (Type 2), which were recently promoted to the species level and named PRRSV-1 and PRRSV-2, respectively (Gulyaeva et al., 2017). For both species, the genome is about 15 kb in length and contains at least 11 open reading frames (ORFs). The replicase gene is composed of ORF1a and ORF1b and occupies the 5’-proximal three-quarters of the viral genome. It encodes two large nonstructural polyproteins, pp1a and pp1ab, with the expression of the latter depending on -1 programmed ribosomal frameshift in the short ORF1a/ORF1b overlap region. The PRRSV pp1a and pp1ab precursors are processed into at least 14 functional nonstructural proteins (nsps) by a complex proteolytic cascade that is directed by four ORF1a-encoded protease domains: three papain-like proteases (PLP1α and PLP1β in nsp1α and nsp1β, respectively, and PLP2 in the N-terminal region of nsp2) and a chymotrypsin-like serine protease (SP) located in nsp4. PLP1α, PLP1β, and PLP2 cleave the junctions between nsp1α/1β, nsp1β/2, and nsp2/3, respectively, thus mediating the rapid release of nsp1α, nsp1β, and nsp2 from the nascent polyproteins (Fang and Snijder, 2010). The nsp2 is the largest viral protein, and previous studies suggest that, in addition to its functions directly related to viral replication, it also serves as an innate immune antagonist (Beura et al., 2010, Frias-Staheli et al., 2007, Sun et al., 2010, Sun et al., 2012, van Kasteren et al., 2012). Frias-Staheli et al. (2007) first demonstrated that PRRSV-2 nsp2 exhibits general deubiquitinase (DUB) activity towards cellular ubiquitin (Ub) conjugates and showed deISGylation activity to remove the conjugates of the IFN-induced Ub homolog ISG15. The DUB activity of the PRRSV PLP2 domain was further characterized in an *in vitro* expression system for both PRRSV species (van Kasteren et al., 2012). The de-ISGylation activity of the PRRSV-1 PLP2 domain was observed in both *in vitro* expression system and infected porcine alveolar macrophages (Sun et al., 2012), although the level of de-ISGylation activity of purified PRRSV-2 PLP2 needs to be evaluated in more detail (Deaton et al., 2014). The biological significance of these activities was supported by the ability of PLP2 to inhibit type I IFN activation and antagonize the antiviral effect of ISG15 (Beura et al., 2010, Sun et al., 2012, van Kasteren et al., 2012).

Recently, in all arteriviruses except for EAV, a new ORF was discovered that overlaps the nsp2-coding region of ORF1a in the –2/+1 reading frame (Fang et al., 2012). This ORF is translated via a unique –2 programmed ribosomal frameshift (PRF) mechanism, which produces a previously unknown transframe product (nsp2TF) consisting of approximately the N-terminal two-thirds of nsp2 and a unique C-terminal extension that is specified by the novel TF ORF (Fang et al., 2012). Remarkably, the same frameshift site was also found to direct an efficient -1 PRF, which is followed by a stop codon, thus yielding a second truncated nsp2 variant named nsp2N (Fang et al., 2012, Li et al., 2014). Our recent work demonstrated that efficient –2 and –1 PRF at this site in the nsp2-coding region depends on the transactivation of frameshifting by the upstream replicase subunit nsp1β, which is thought to bind together with cellular poly(C) binding proteins to the genomic region containing the –2/–1 PRF signal, possibly to form a roadblock for the translating ribosome (Li et al., 2014, Napthine et al., 2016).

The newly identified nsp2TF and nsp2N proteins add to the functional complexity of the nsp2 region of the viral replicase, a region that has also been explored in the context of the development of genetically modified live virus (MLV) vaccines [reviewed in (Fang and Snijder, 2010, Lunney et al., 2016)]. Importantly, nsp2, nsp2TF, and nsp2N all include the N-terminal PLP2 domain, which has been implicated in disrupting type I interferon signaling by deubiquitination and deISGylation of cellular proteins, as outlined above. In this study, we analyzed the effect of nsp2TF and nsp2N expression on host innate immune responses, both in an *in vitro* expression system and using recombinant viruses with impaired nsp2TF/nsp2N expression. An immune gene mRNA profiling system was employed to analyze the expression of a predefined set of 579 immune genes in cells infected with wild-type or nsp2TF/nsp2N-deficient viruses. A panel of innate immune genes was found to be upregulated in cells infected with nsp2TF/nsp2N-deficient viruses. Subsequent *in vivo* studies consistently showed that nsp2TF/nsp2N-deficient viruses were less capable of interfering with the innate immune response in infected pigs. These studies provide important insights into the potential role(s) of PRRSV nsp2TF and nsp2N in the modulation of host innate immune responses.

## 2 Results

### 2.1 In vitro expression of PRRSV nsp2TF or nsp2N affects cellular innate immune responses

**Fig. 1:**
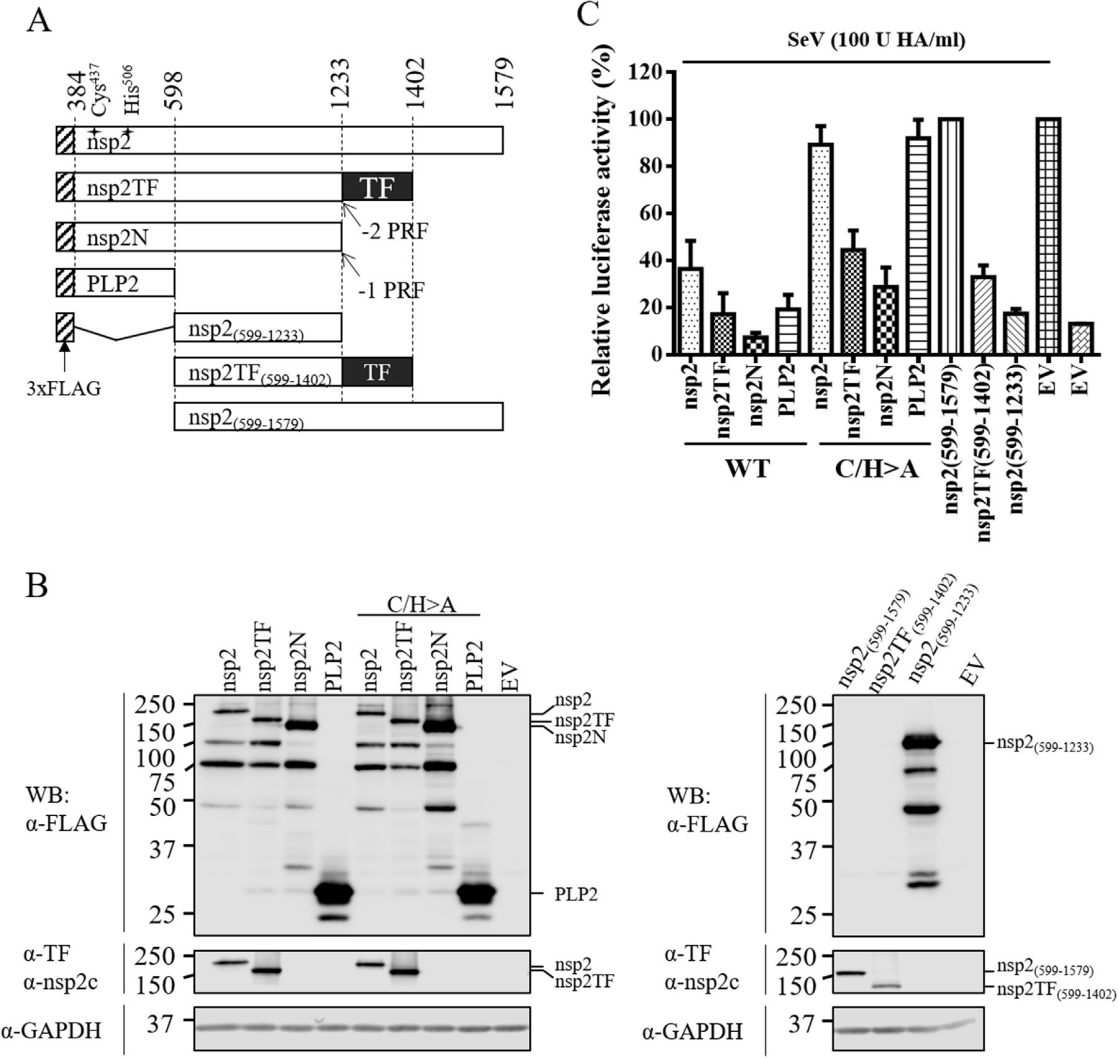
**PRRSV nsp2 variants suppress type I IFN production.** (A) A schematic diagram of individual PRRSV nsp2 variants (nsp2, nsp2TF and nsp2N) and domains [PLP2, nsp2_(599–1233)_, nsp2TF_(599–1402)_ and nsp2_(599–1579)_]. The numbers show both N- and C-terminal residues on polyprotein 1a for each individual protein. The catalytic residues of the PLP2 domain are labeled as Cys^437^ and His^506^. (B) Expression of nsp2-related proteins were evaluated by western blot analysis using anti-FLAG M2 mAb. GAPDH was detected as loading control. Rabbit pAb against the unique nsp2 C-terminus was used to confirm the expression of nsp2 and nsp2_(599–1579)_, while rabbit pAb against unique nsp2TF C-terminus was used to confirm the expression of nsp2TF and nsp2TF_(599–1402)_. (C) Effect of nsp2-related proteins on the expression of IFN-β promoter-driven luciferase expression. HEK-293T cells were cotransfected with a plasmid expressing individual nsp2-related proteins or domains, p125-Luc reporter plasmid expressing firefly luciferase under the control of the IFN-β promoter (0.5 μg), and pRL-SV40 reporter plasmid (20 ng). An empty vector (EV) was used as a control. At 24 h posttransfection, cells were stimulated with SeV at 100 HA units/mL or for 16 h. Cell lysates were harvested for measuring luciferase activity. Relative luciferase activities were calculated by normalizing the firefly luciferase to *Renilla* luciferase activities; the relative luciferase activity in cells with EV-transfection and SeV stimulation was set as 100%. The mean value and SEM of representative experiments are shown. All experiments were repeated at least three times, and duplicates were performed each time.

**Fig S1:**
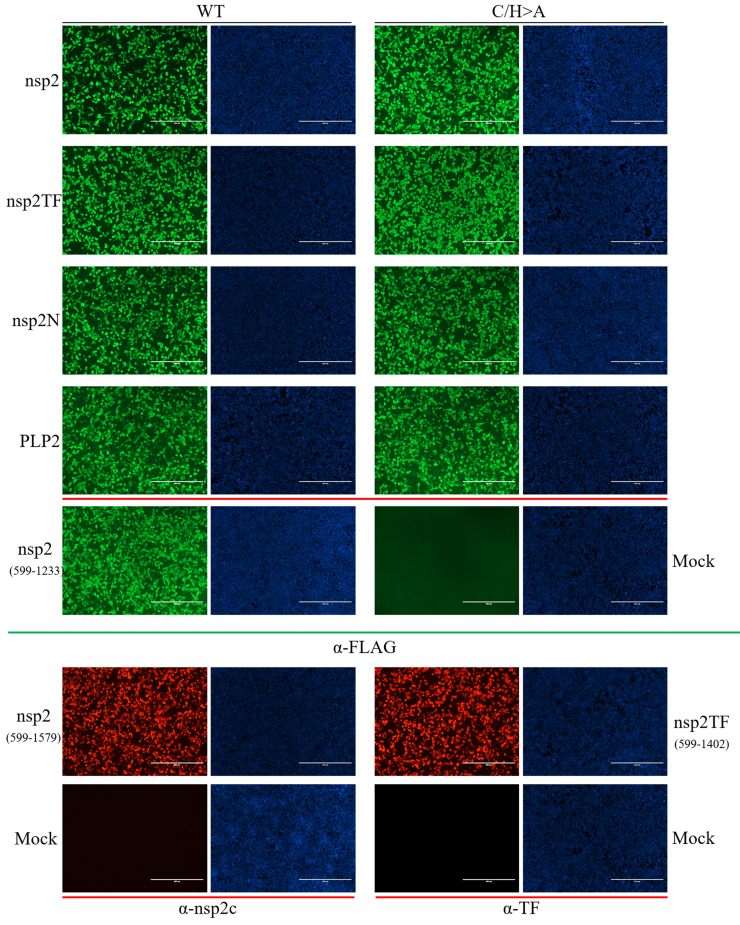
**Expression of nsp2-related proteins in HEK-293T cell**. HEK-293T cells seeded in a 24-well plate were transfected with a plasmid (0.5 μg) expressing each of nsp2-related protein. At 24 hpi, immunofluorescence assay was performed to evaluate protein expression. The α-FLAG mAb was used to detect the expression of FLAG-tagged proteins. Rabbit pAb (α-TF) specifically recognizing the unique C-terminal domain of nsp2TF was used to detect the expression of full-length nsp2TF (Li et al., 2014), while rabbit pAb (α-nsp2) specifically recognizing the nsp2C-terminus was used to detect the expression of full-length nsp2 (Guo et al., 2016). Cell nucleus was stained with DAPI.

**Fig S2:**
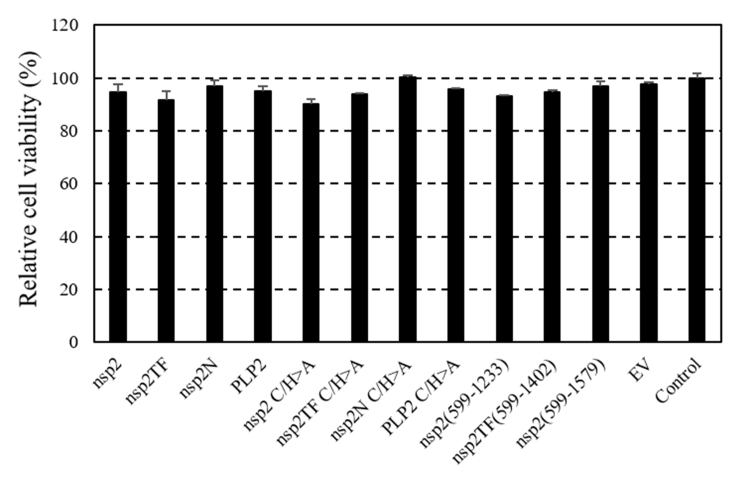
**Effect of nsp2-related proteins on HEK-293T cell viability.** Cell viability was determined using a CellTiter 96 AQ_ueous_ cell proliferation assay (Promega). Viability of transfected cells were compared to that of untreated control cells (100%). The average and standard deviation (SD) of a representative experiment are shown. All experiments were repeated twice, and triplicates were performed at each time.

To investigate the innate immune suppression capability of nsp2TF and nsp2N, we expressed them individually in the context of a luciferase reporter assay, which is based on the expression of a firefly luciferase reporter gene under the control of an IFN-β promoter (Yoneyama et al., 1996). IFN-β signaling was activated by infection with Sendai virus and the luciferase expression level was measured at 16 h after stimulation. PRRSV sequences (PRRSV-2, strain SD95-21) encoding full-length nsp2, nsp2TF, or nsp2N were expressed as an N-terminally FLAG-tagged fusion protein using a eukaryotic expression vector (Fig. 1A). The empty vector (EV) and a plasmid expressing the FLAG-tagged PLP2 domain, pFLAG-PLP2, were included as negative and positive controls, respectively. Similar transfection rate of ~80% was confirmed by immunofluorescence assay in HEK-293T cells transfected with these expression constructs (Fig. S1). No obvious cytotoxic effects of protein expression were observed in transfected cells as determined by cell viability assay (Fig. S2). Protein expression was evaluated by western blot analysis (Fig. 1B). Of note, many nsp2-related proteins of smaller size (less than 100 kDa) were detected using the antibody (M2) recognizing the N-terminal FLAG-tag, yielding a similar pattern (with some exceptions) for the constructs expressing nsp2, nsp2TF, and nsp2N. This suggested that these products are C-terminally truncated expression products of distinct size.

In line with previous studies (Sun et al., 2010, Sun et al., 2012, van Kasteren et al., 2012), expression of PLP2 strongly suppressed luciferase reporter gene expression from the IFN-β promoter following SeV infection. Expression of nsp2, nsp2TF, and nsp2N showed inhibitory effects on the IFN-β promoter activity, as indicated by around 60%, 80%, and 90% reduction of luciferase activity, respectively, in comparison to EV-transfected control cells (Fig. 1C). Next, to assess whether these inhibitory effects were directly linked to PLP2's protease/DUB activity, we engineered a panel of constructs in which the PLP2 catalytic residues Cys^437^ and His^506^ were both substituted with alanine (C/H>A). In HEK-293T cells transfected with these constructs, nsp2-related proteins were expressed (Fig. 1B). Most of the nsp2-related proteins of smaller size were also observed, suggesting these proteins did not result from PLP2-mediated cleavage of the expression products. Surprisingly, expression of nsp2TF-C/H>A and nsp2N-C/H>A still significantly inhibited IFN-β signaling following SeV stimulation (about 50% and 70% reduction compared to EV-transfected control cells). In contrast, in the context of the expression of full-length nsp2 or the PLP2 domain only, introduction of the PLP2-C/H>A mutation strongly impaired its ability to inhibit luciferase expression (Fig. 1C). Compared to the PLP2 construct, nsp2TF and nsp2N contain an additional 804 amino acids (aa 599–1402) and 635 amino acids (aa 599–1233), respectively. Our data suggested that, besides the PLP2/DUB domain, the downstream sequences of nsp2TF [nsp2TF_(599–1402)_, see Fig. 1A] and nsp2N [nsp2_(599–1233)_, see Fig. 1A] could also contain activities that antagonize innate immune signaling. Consistent with this hypothesis, the ectopically expressed nsp2TF_(599–1402)_ and nsp2_(599–1233)_ exhibited strong inhibitory effects on IFN-β promoter activity (about 70% and 80% reduction) in comparison to that of EV control (Fig. 1C).

**Fig. 2:**
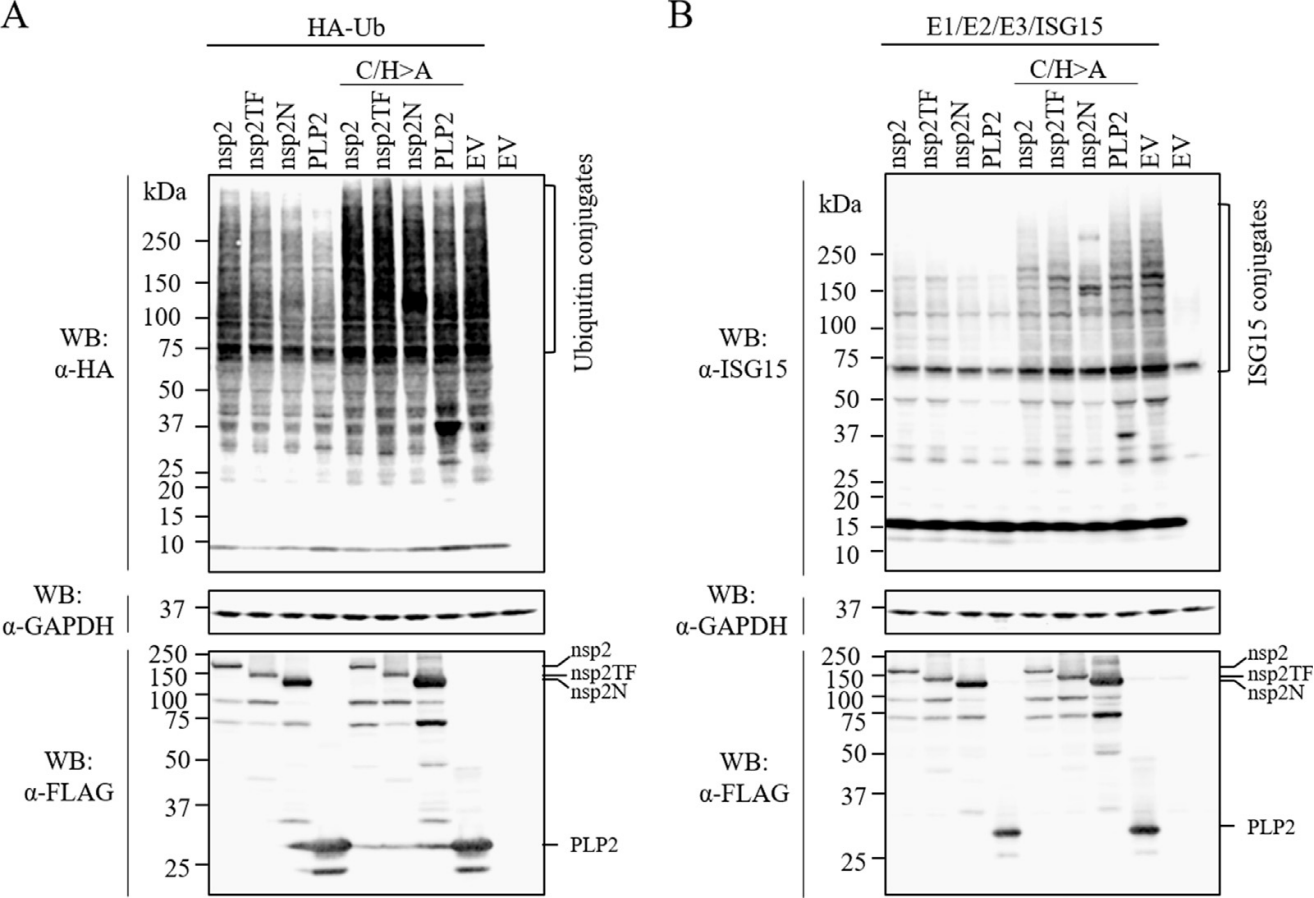
**The de-ubiquitination and de-ISGylation activities of PRRSV nsp2 related proteins.** (A) Effect of nsp2 related protein expression on ubiquitin conjugation. HEK-293T cells were co-transfected with plasmid DNAs expressing HA-Ub and FLAG-tagged nsp2, nsp2TF, nsp2N, PLP2 or their catalytic site mutants (nsp2-C/H>A, nsp2TF-C/H>A, nsp2N-C/H>A, PLP2-C/H>A). HA-Ub conjugated cellular proteins were visualized by western blot analysis using anti-HA mAb. The expression of FLAG-tagged nsp2 related proteins was detected by anti-FLAG M2 mAb. (B) HEK-293T cells were co-transfected with plasmid DNAs expressing ISG15 and its conjugation enzymes E1/E2/E3, FLAG-tagged nsp2, nsp2TF, nsp2N, PLP2 or their catalytic site mutants (nsp2-C/H>A, nsp2TF-C/H>A, nsp2N-C/H>A, PLP2-C/H>A). ISG15-conjugated cellular proteins were detected by ISG15 specific mAb in western blot analysis. The expression of FLAG-tagged nsp2 related proteins was detected by anti-FLAG M2 mAb. For both assays, the empty p3xFLAG vector plasmid was included as a control, while expression of housekeeping gene GAPDH was detected as a loading control.

Since PLP2 was reported to also act as a deubiquitinase (Deaton et al., 2014, Frias-Staheli et al., 2007, Sun et al., 2010, van Kasteren et al., 2012), we further compared the effect of full-length nsp2, nsp2TF, and nsp2N on host cell protein ubiquitination. HEK-293T cells were transfected with a plasmid expressing HA-tagged ubiquitin and a plasmid expressing FLAG-tagged full-length nsp2, nsp2TF, or nsp2N. Again, the plasmid expressing the PLP2 domain and the empty vector were used as controls. As shown in Fig. 2A, expression of all four PLP2-containing products resulted in a decreased level of ubiquitin-conjugated proteins, but compared to full-length nsp2 and nsp2TF, nsp2N had a stronger effect and its DUB ability was comparable to that of the PLP2 domain. Subsequently, we analyzed the effect of nsp2TF and nsp2N expression on cellular protein ISGylation. In HEK-293T cells, ISG15 conjugates were generated by co-transfecting plasmids expressing ISG15 and three conjugation enzymes E1, E2, and E3. Cells were co-transfected with plasmids expressing one of the individual nsp2-related proteins. Co-expression of either nsp2TF or nsp2N resulted in a clear decrease in the level of ISGylated cellular proteins (Fig. 2B). To directly link the DUB and deISGylation activity to the PLP2 domain of these proteins, we repeated the assays using the respective C/H>A mutants. As shown in Fig. 2, compared to cells expressing the wild type nsp2-related proteins, much higher levels of ubiquitin and ISG15 conjugation were detected in cells expressing any of the corresponding C/H>A mutants. Taken together, these data revealed that individually expressed nsp2, nsp2TF, and nsp2N have the ability to interfere with cellular protein ubiquitination and ISGylation processes to variable degrees, with nsp2N showing the strongest effect. Moreover, the deconjugating activities of these nsp2-related proteins can be impaired by inactivating their PLP2 activity.

### 2.2 Construction and characterization of in vitro properties of nsp2TF/nsp2N-deficient mutants

**Fig. 3:**
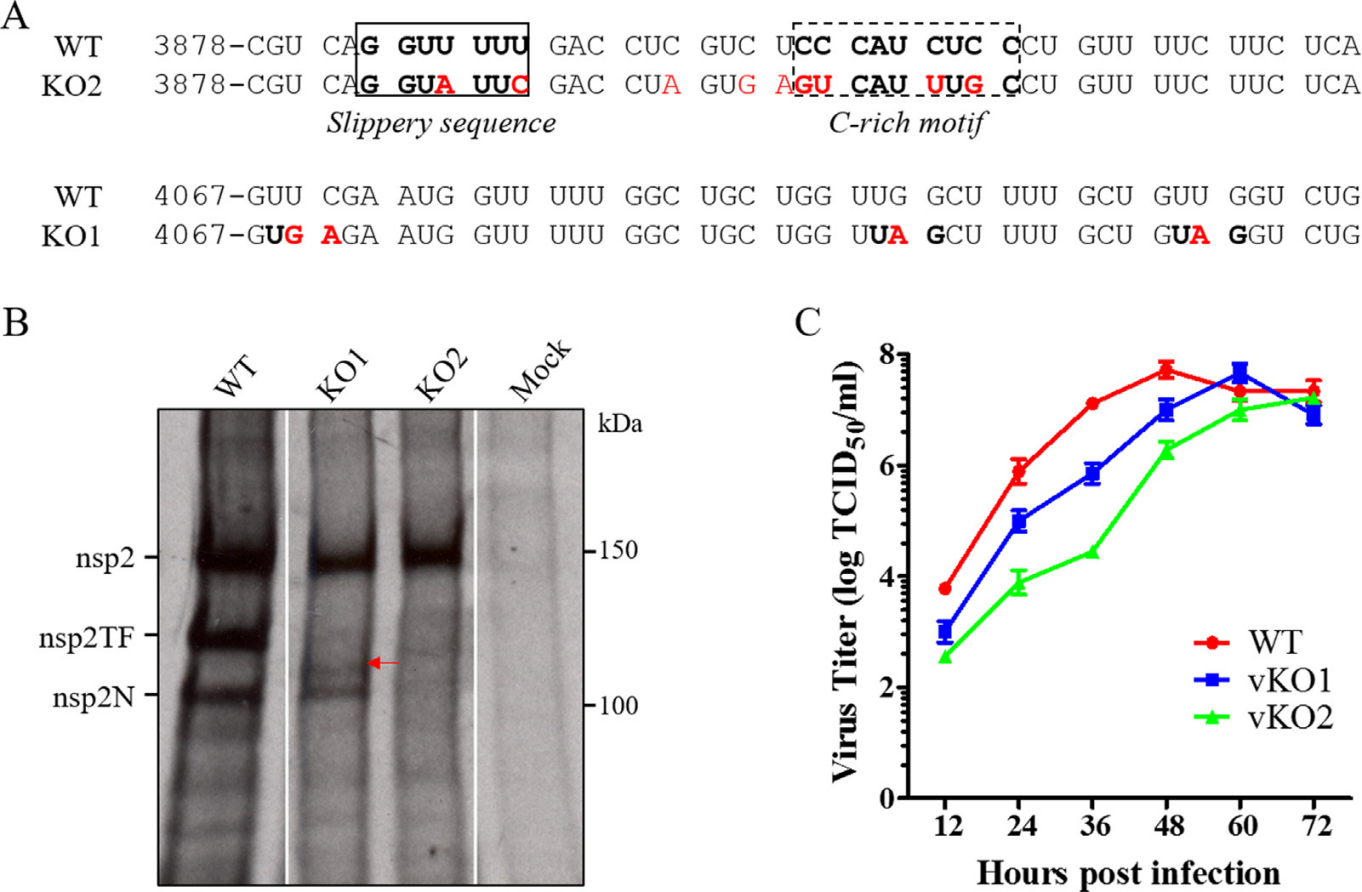
**Construction and*****in vitro*****characterization of nsp2TF/nsp2N-deficient mutants.** (A) Nucleotide sequences in the regions of mutations created in nsp2TF/nsp2N-deficient mutants. KO1, knockout mutant 1 (premature termination codons in TF ORF); KO2, knockout mutant 2 (premature termination codon and disrupted frameshift signal). Mutated nucleotides are shown in red. (B) Radioimmunoprecipitation detection of nsp2-related proteins in virus infected cells. MAb 140-68 was used to recognize the common N-terminal PLP2 domain. The truncated nsp2TF in vKO1-infected cells is indicated with a red arrow. (C) Multiple-step virus growth curve. Each data point shown represents the mean value from duplicates, and error bars show SEM.

To determine whether the immune suppression potential of nsp2TF and nsp2N observed in *in vitro* expression systems reflect their actual function in PRRSV-infected cells, we employed a previously described strategy to create PRRSV-1 and PRRSV-2 mutants that are partially or completely deficient in expression of nsp2TF/nsp2N (Fang et al., 2012, Li et al., 2014). For the present study, PRRSV-2 mutants were analyzed, in which -2/-1 PRF-inactivating mutations were engineered in the full-length cDNA clone of PRRSV-2 strain SD95-21 (Fig. 3A). Mutant vSD95-21-KO1 (vKO1) retained an intact PRF signal, meaning that the relative translation of the three different ORFs should not be affected. However, whereas production of nsp2 and nsp2N was unchanged, nsp2TF was truncated due to the insertion of three stop codons in the TF ORF, resulting in the production of a C-terminally truncated form of nsp2TF (98.5 kDa, indicated by a red arrow in Fig. 3B). Mutant vSD95-21-KO2 (vKO2) carried a combination of mutations that disrupt the PRF slippery sequence and the downstream PRF-stimulatory CCCANCUCC motif. These mutations were previously shown to fully inactivate the PRRSV -2/-1 PRF without affecting nsp2 expression, since all mutations used were translationally silent with respect to ORF1a (Fang et al., 2012, Li et al., 2014). As shown in Fig. 3B, the expression of nsp2 appeared not to be affected in MARC-145 cells infected with either of these mutants. In comparison to the WT virus in MARC-145 cells, replication of vKO1 and vKO2 mutants was impaired, with 1- to 2-log decreased virus titers before 48 hpi. The peak titer of vKO2 was 0.5-log lower than that of WT virus, while vKO1 reached a similar peak titer as WT virus 12 h later (at 60 hpi, Fig. 3C).

### 2.3 Cellular immune gene expression is strongly upregulated in cells infected with nsp2TF/nsp2N-deficient PRRSV

**Fig. 4:**
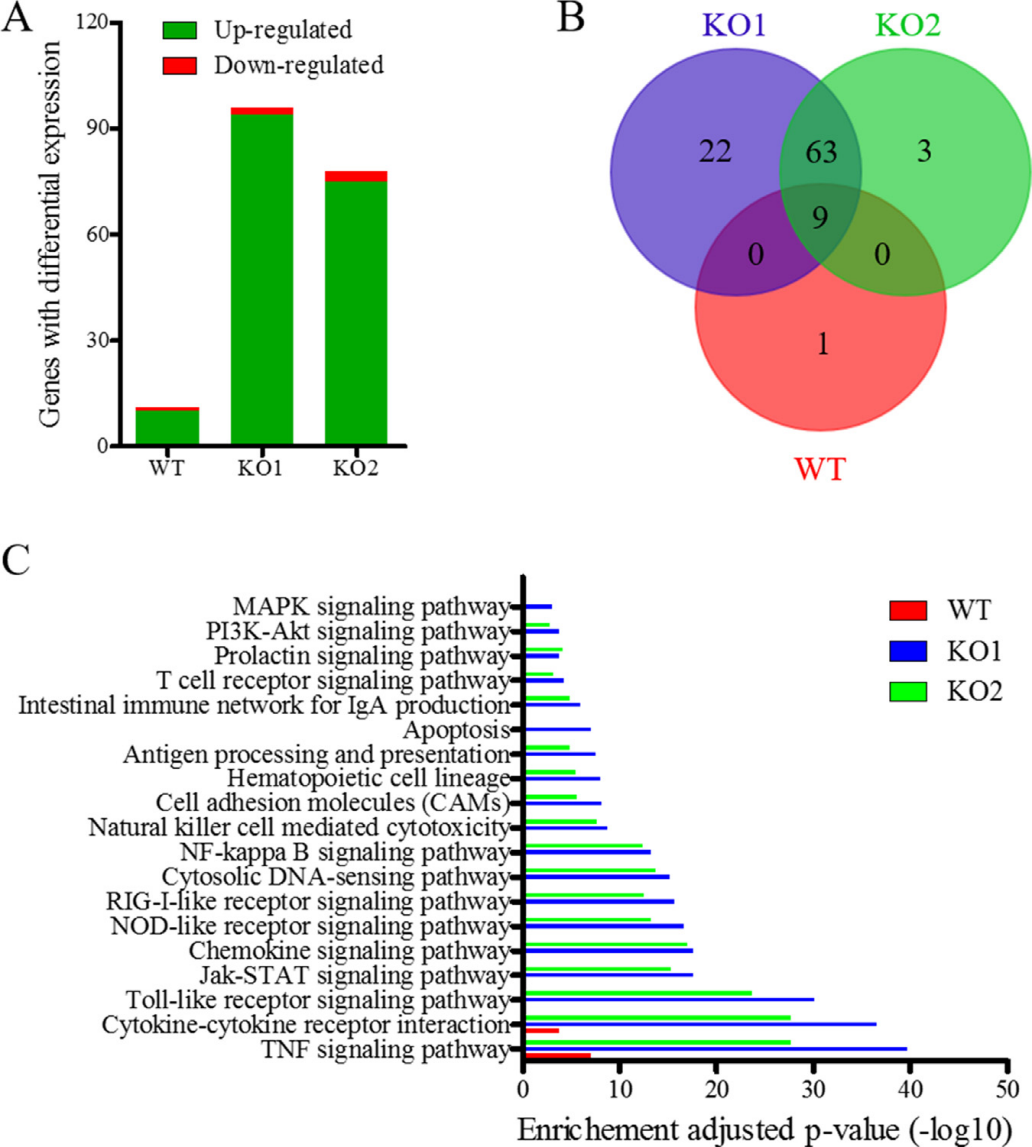
**nCounter mRNA Profiling of immune gene expression in virus-infected cells.** (A) An overview on the number of differentially expressed genes (DEG) in virus-infected cells at 12 hpi. (B) Common and uniquely upregulated DEGs in virus-infected cells at 12 hpi. (C) Enriched functional categories of DEGs in virus-infected cells at 12 hpi determined by KEGG Pathway Enrichment analysis using DAVID (Huang et al., 2009a, Huang et al., 2009b). P-values adjusted by Benjamini-Hochberg correction less than 0.05 were defined as significant enrichment. The x-axis is the negative log10 of P-value.

To investigate whether impaired nsp2TF/nsp2N expression would alter the ability of PRRSV to interfere with cellular immune gene expression, an immune gene mRNA expression profile was determined for MARC-145 cells (a cell line of African green monkey origin) infected with WT PRRSV-2 strain SD95-21 and mutants vKO1 and vKO2. Since there is no monkey gene specific nCounter kit commercially available, we used the nCounter system that was designed for simultaneously analyzing the expression of a predefined set of 579 human immune genes (Human_Immunology_v2_Panel; NanoString Technologies, Seattle, WA). In comparison to mock-infected cells, a total of 11 differentially expressed genes (DEGs) were identified in WT virus-infected cells, while 96 and 78 DEGs were identified in cells infected with vKO1 and vKO2, respectively (Fig. 4A, Table S1). Among these hits, 10, 94, and 75 DEGs were upregulated in cells infected with WT virus, vKO1, and vKO2, respectively. Venn diagram analysis showed that the expression of 9 immune genes was upregulated in cells infected with all three viruses, whereas 63 DEGs were upregulated only in cells infected with either vKO1 or vKO2, and 1, 22, and 3 DEGs were specifically upregulated in cells infected with WT virus, vKO1, and vKO2, respectively (Fig. 4B, Table S3). In comparison to WT virus, vKO1 and vKO2 infection upregulated the expression of 84 and 65 more immune genes in host cells, respectively. To identify biological pathways associated with the identified DEGs, we performed a KEGG pathway enrichment analysis using the DAVID program (Huang et al., 2009a, Huang et al., 2009b) to identify pathways that were consistently enriched in target cells (Fig. 4C, Table S2). The cytokine and cytokine receptor interaction and TNF signaling pathway were enriched pathways in WT virus-infected cells. By contrast, there were many additional pathways enriched in cells infected with vKO1 and vKO2, and the most strongly activated pathways identified were those involved in cytokine-cytokine receptor interaction, TNF signaling, Toll-like receptor signaling, NOD-like receptor signaling, NF-κB signaling, RIG-I-like receptor signaling, chemokine signaling, JAK-STAT signaling, cytosolic DNA-sensing, and natural killer cell mediated cytotoxicity. Most of these strongly activated pathways are involved in the host cell's innate immune response.

**Fig S3:**
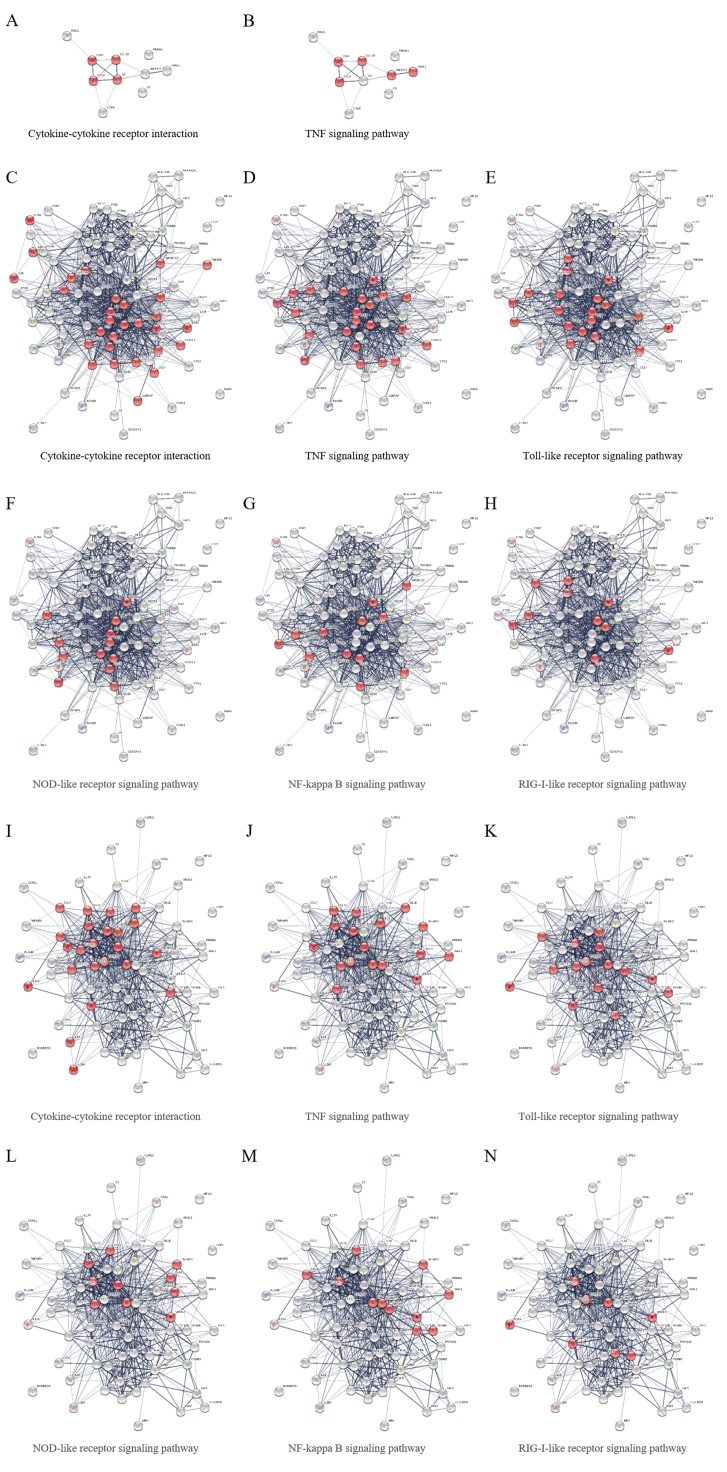
**Protein-protein interaction networks of DEGs in cells infected with WT virus and nsp2TF/nsp2N-deficient mutants.** Protein-protein interaction networks were constructed for all DEGs in cells infected with WT virus (A-B), vKO1 (C-H), and vKO2 (I-N) using the search tool for the retrieval of interacting genes/proteins (STRING). Confidence view of protein-protein interaction network from DEGs showing the strength of data support is indicated by the thickness of the grey line connecting genes and nodes. The representative pathways enriched in cells infected with WT virus (A-B), vKO1 (C-H), and vKO2 (I-N) were highlighted with red color.

To better understand the DEGs and visualize relevant activated pathways, the protein-protein interactions (PPI) networks of DEGs identified in infected cells were generated using the Search Tool for the Retrieval of Interacting Genes/Proteins (STRING), which is a database that provides a critical assessment and integration of protein–protein interactions, including direct (physical) as well as indirect (functional) associations (Szklarczyk et al., 2015). Consistent with the KEGG pathway enrichment analysis using DAVID, all enriched pathways in vKO1- and vKO2-infected cells identified above were significantly activated in PPI networks, while only cytokine-cytokine receptor interaction and TNF signaling pathways were significantly activated pathways in WT virus-infected cells. The representative enriched pathways in cells infected with WT, KO1 virus, and KO2 virus were highlighted in the protein-protein interactions networks of DEGs, respectively (Fig. S3).

**Fig. 5:**
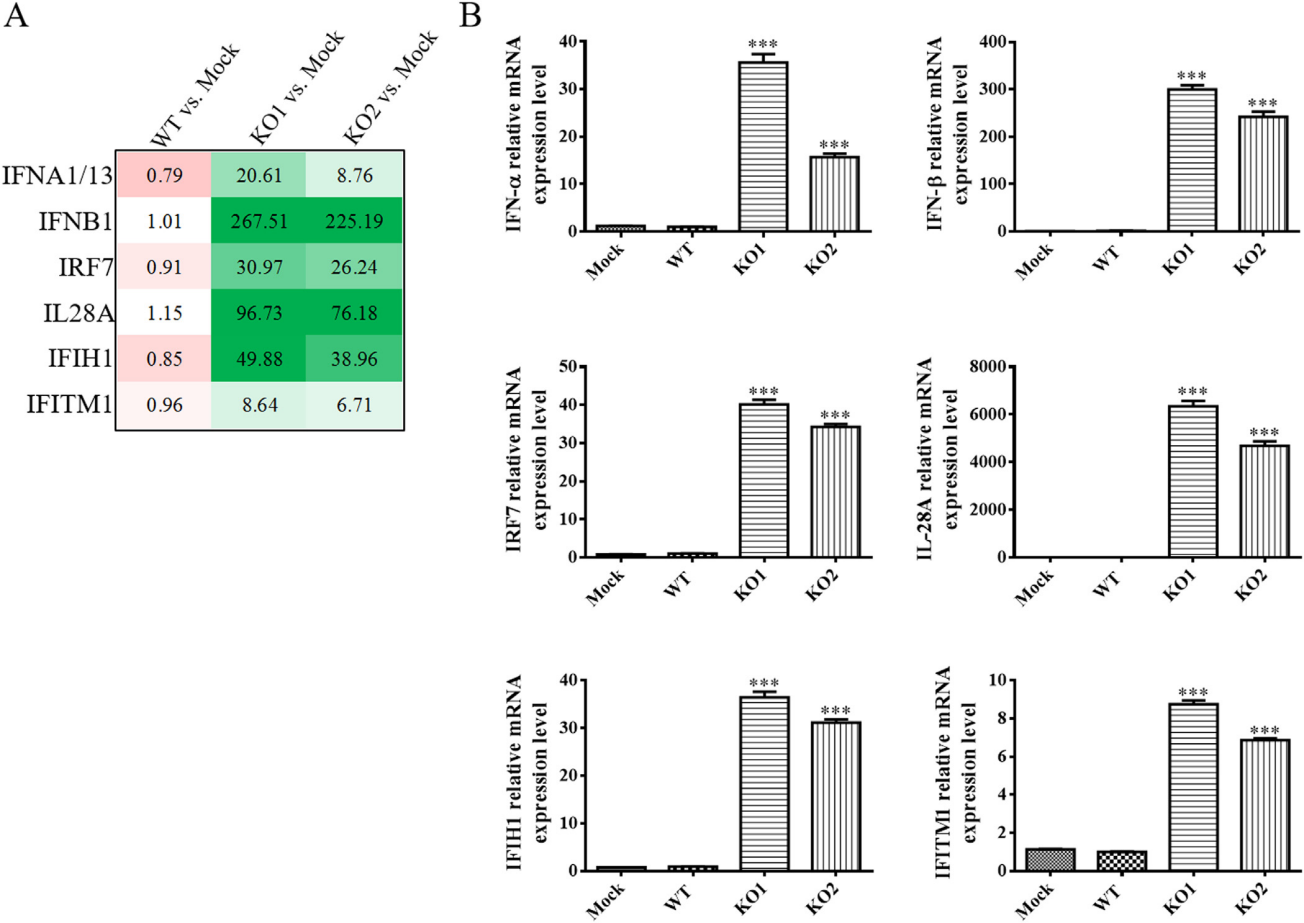

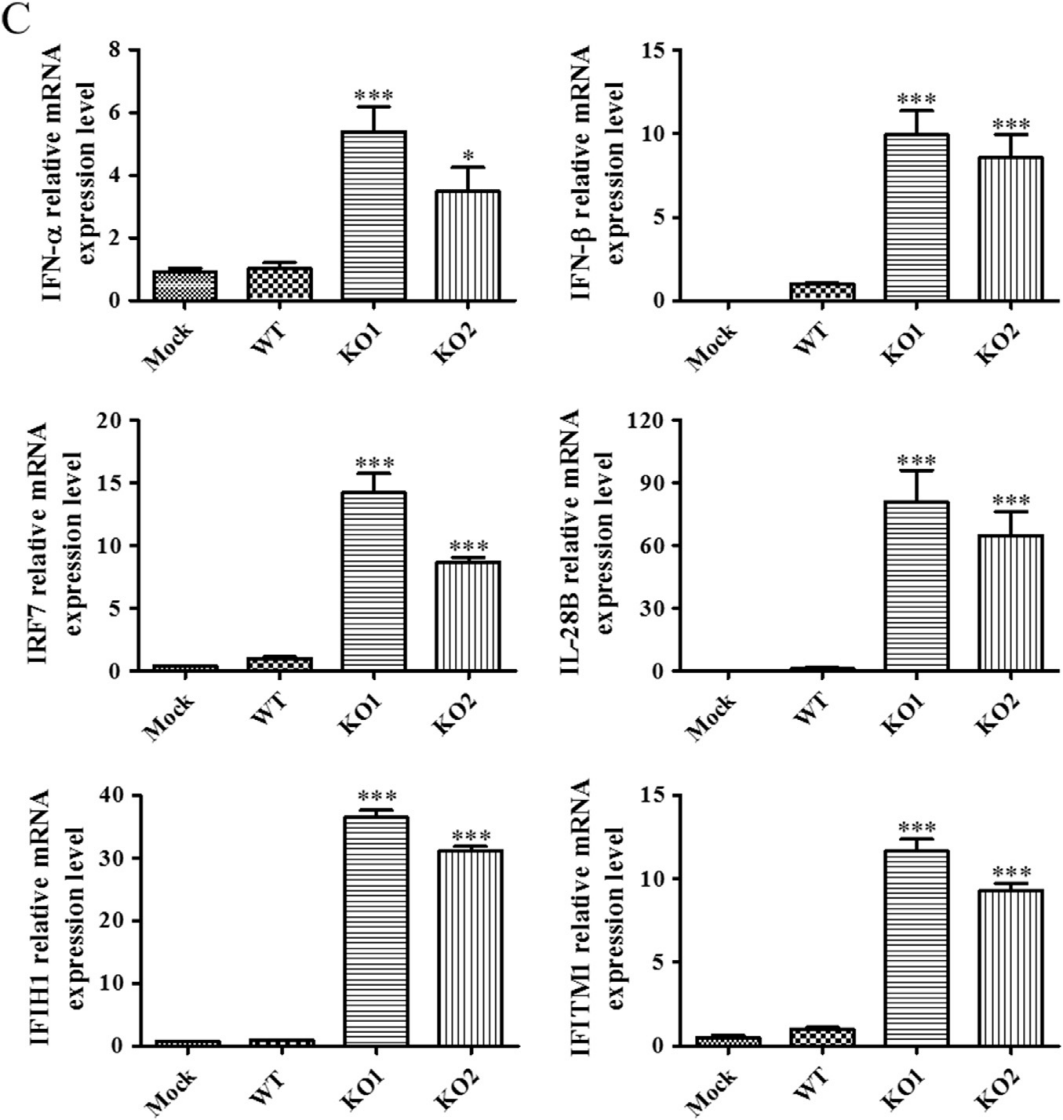
**Quantitative RT-PCR detection of the expression of selected differentially expressed genes in virus-infected cells.** (A) Six selected immune genes with increased expression levels in cells infected by nsp2TF/nsp2N-deficient mutants. Each data point shown represents the mean value from five replicates, and the gene expression levels in mock were treated as 1. (B) The relative expression levels of six immune genes in virus-infected MARC-145 cells at 12 hpi. (C) The relative expression levels of six immune genes in virus-infected PAM at 12 hpi. (B-C) Each data point shown represents the mean value from five replicates, and the gene expression levels in WT infected cells were treated as 1.

The above data analysis showed that most of the pathways activated by vKO1 and vKO2 infection are involved in the host's innate immune response. Next, we performed quantitative RT-PCR (qRT-PCR) to independently verify the gene expression levels of six representative innate immune genes: IFN-α, IFN-β, IRF7, IL-28A, IFIH1, and IFITM1. Based on the analysis using the nCounter system, these genes had significantly higher expression levels in vKO1- and vKO2-infected cells than in WT virus-infected cells (Fig. 5A). Using the same RNA samples for multiplex digital mRNA profiling, the expression levels of these genes were evaluated with commercially available TaqMan Gene Expression assays (Thermofisher Scientific, Waltham, MA), and their relative expression levels were calculated. The TaqMan probes were specifically designed for the targeted monkey genes. As shown in Fig. 5B, the relative gene expression levels of the six innate immune genes generally were highly consistent with the data generated using the nCounter system (Fig. 5A).

To further confirm the results generated using nCounter and TaqMan analysis, the ability of WT and mutant viruses to stimulate the expression of this panel of representative genes was verified in porcine alveolar macrophages (PAM), the primary target cell of PRRSV in infected animals. PAM cells were infected with the WT and mutant viruses at an MOI of 1, and harvested at 12 hpi. Swine gene specific qRT-PCR assays were used to analyze the expression levels of the selected immune genes. Consistent with the increased expression levels detected in MARC-145 cells, vKO1 and vKO2 induced significantly higher expression levels of IFN-α, IFN-β, IRF7, IL-28B, IFIH1, and IFITM1 in PAM cells (Fig. 5C).

### 2.4 Effect of nsp2TF/nsp2N deficiency on innate and cell-mediated immune responses in PRRSV-infected pigs

**Fig. 6:**
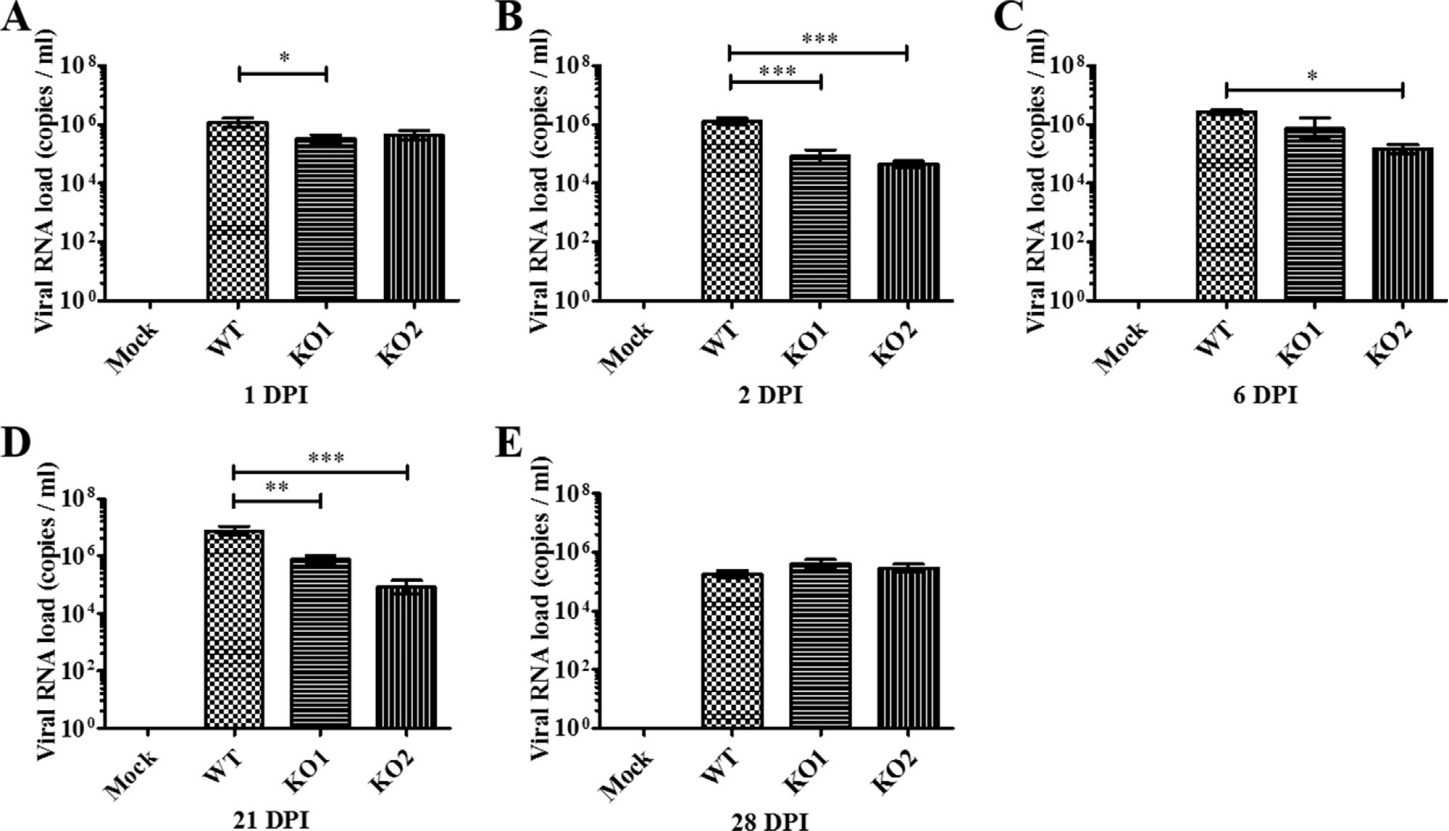
**Comparison of viral RNA loads in serum samples from pigs inoculated with the WT virus and nsp2TF/nsp2N-deficient mutants.** Pigs were uninfected (mock) or infected with WT PRRSV (WT), vKO1 or vKO2 mutant. Serum samples were collected on the indicated days post-infection. Viral loads in serum samples quantified by quantitative RT-PCR and calculated as viral RNA copies per milliliter. Statistical significance between the WT virus-infected group and mutant virus-infected groups was determined by one-way ANOVA (Tukey's test) and indicated with asterisks (*, P<0.05; **, P<0.01; ***, P<0.001).

To assess whether the *in vitro* data described above could be reproduced in PRRSV-infected pigs, we investigated the impact of nsp2TF/nsp2N-deficient mutations on innate immune responses in PRRSV-infected nursery pigs. Four groups of pigs (n=9) were used, which were infected with WT virus (group 1), vKO1 (group 2), vKO2 (group 3), while the negative control group (group 4) was kept uninfected. To verify that WT and both mutant viruses replicated *in vivo*, serum samples collected by 6 days post infection (DPI) were used for virus isolation on MARC-145 cells. Virus was recovered from the serum samples of the pigs in groups 1–3, indicating active replication of WT and mutant viruses in pigs. The mutations introduced into the vKO1 and vKO2 genomes were found to be genetically stable throughout the study, as determined by sequencing of RT-PCR products derived from serum samples of vKO1- and vKO2-infected pigs at 21 dpi. Viral load in serum samples collected at 1, 2, 6, 21, 28 DPI was further quantified by qRT-PCR. The results showed reduced viral loads in pigs infected with vKO1 and vKO2, which is consistent with the observations made in cell culture (Fig. 3C). At 1, 2 and 21 DPI, the group of pigs infected with vKO1 had significantly lower viral load (0.5–1.2 logs lower) than those pigs infected with WT virus (Fig. 6A, B and D). At 2, 6 and 21 DPI, viral loads in serum of vKO2-infected pigs were about 0.9–2 logs lower than those of WT virus-infected pigs (Fig. 6B-D).

**Fig. 7:**
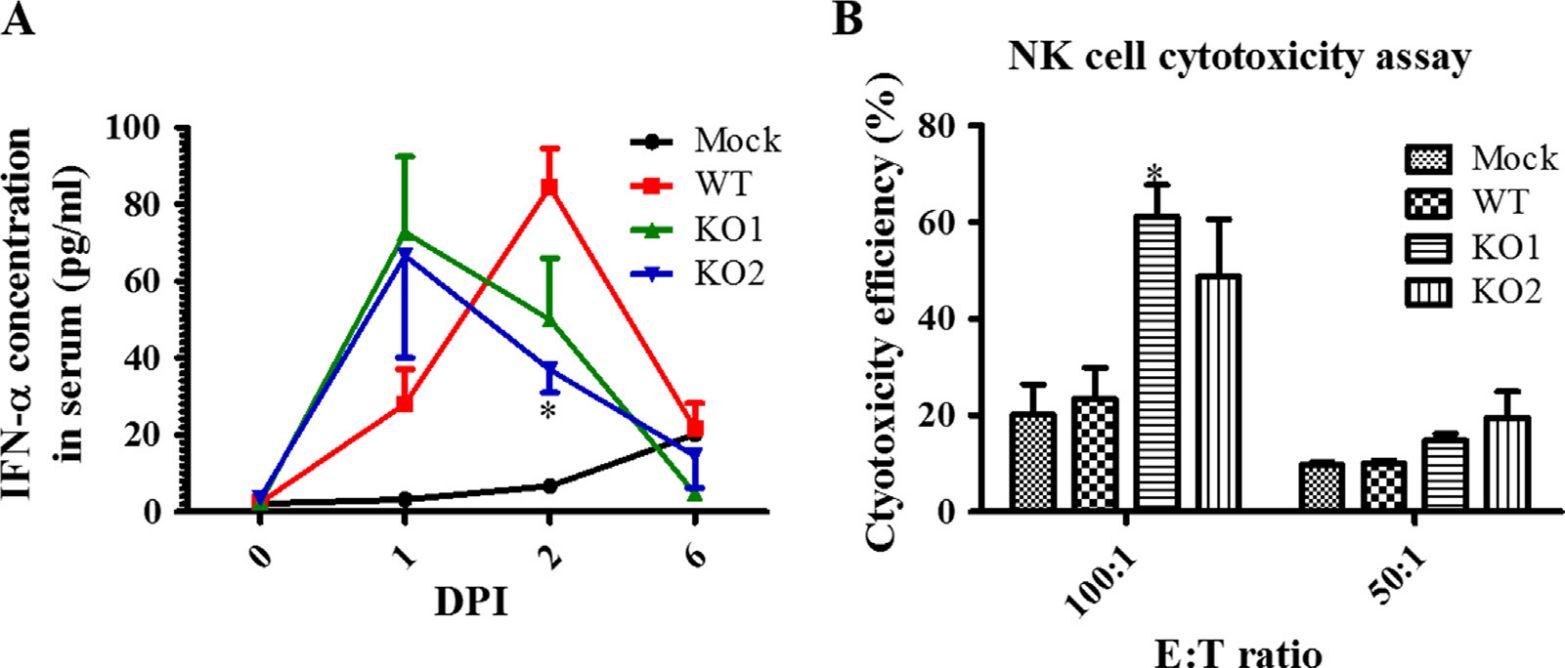
**Comparison of IFN-α production levels and NK cell cytotoxicity in pigs inoculated with WT virus and nsp2TF/nsp2N-deficient mutants**. Pigs were uninfected (mock) or infected with WT PRRSV (WT), vKO1 or vKO2 mutant. (A) IFN-α levels in serum samples collected at 0, 1, 2 and 6 DPI were analyzed with a ProcartaPlex Porcine IFN alpha Simplex kit. (B) PBMCs (NK effectors) harvested on the day of necropsy (6 DPI) were co-cultured with target cells (K562) at an E:T ratio of 100:1 or 50:1. After overnight incubation, flow cytometry was performed to evaluate the NK cell-specific cytotoxic activity. Each data point represents the mean value and SEM of data from 3 pigs. Statistical significance between the wild-type virus infected group and mutant virus-infected groups was determined by one-way ANOVA (Tukey's test) and indicated with asterisks (*, P<0.05; **, P<0.01; ***, P<0.001).

To assess innate immune responses, IFN-α production in serum samples at 1, 2 and 6 DPI was initially evaluated with a ProcartaPlex Porcine IFN alpha Simplex kit (eBioscience, San Diego, CA). Compared with WT virus, vKO1 and vKO2 infection induced an earlier IFN-α response (Fig. 7A). Although similar peak concentrations of IFN-α were detected in all three groups of infected pigs, IFN-α level peaked one day earlier (at 1 DPI) in pigs infected with vKO1 and vKO2 compared to pigs infected with WT-virus. Of note, the IFN-α production in pigs correlated well with the viral loads that were measured (Fig. 6). In terms of IFN-α titer and viral load, the largest difference between WT virus and mutant virus infection groups was observed at 2 DPI.

The IFN-α response is critical for natural killer (NK) cell-mediated cytotoxicity. In our nCounter analysis, the genes associated with the NK cell cytotoxicity pathway were enriched in vKO1- and vKO2-infected cells (Fig. 4C; Table S2). Therefore, we further evaluated NK cell-mediated cytotoxicity in our experimentally infected pigs. Peripheral blood mononuclear cells (PBMCs) isolated from pigs infected with vKO1, vKO2 and WT virus were used as a source of NK cells (effectors; E) to evaluate NK cell cytotoxic activity using human myeloblastoid K562 cells as target (T) cells. At both E:T ratios used (100:1 and 50:1), increased NK cell cytotoxicity was observed in PBMCs from vKO1 and vKO2-infected pigs at 6 DPI (Fig. 7B).

**Fig. 8:**
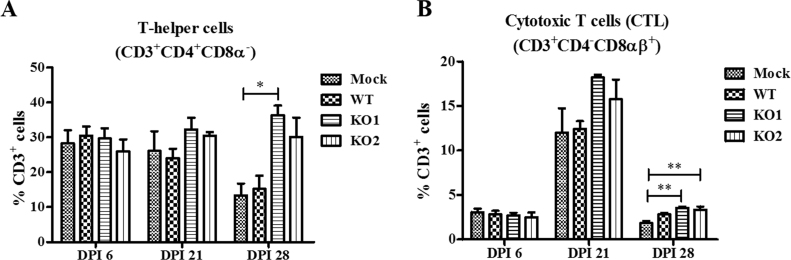
**T-helper and cytotoxic T cell responses in pigs infected with WT virus and nsp2TF/nsp2N-deficient mutants.** PBMCs isolated at 6, 21 and 28 DPI were unstimulated or restimulated with the WT virus. Cells were immunostained for pig specific markers CD3, CD4, CD8α and CD8β, and the frequency of each lymphocyte subset was grouped based on the combination of markers: (A) CD3^+^CD4^+^CD8α^-^ (T-helper cells) and (B) CD3^+^CD4^-^CD8αβ^+^ (cytotoxic T cells) after analysis by flow cytometry. Statistical significance between the groups was determined by one-way analysis of variance (ANOVA) followed by Tukey's post hoc test. (*P<0.05; **P<0.01).

A strong innate immune response following a viral infection is expected to enhance the cell-mediated adaptive immunity (Iwasaki and Medzhitov, 2015, Jain and Pasare, 2017). We further measured the frequency of different T cell subpopulations in PBMCs. Swine T cells expressing the combination of phenotypic markers CD3^+^CD4^+^CD8α^-^ are T-helper cells, and cells with CD3^+^CD4^-^CD8αβ^+^ are exclusively cytotoxic T lymphocytes (CTLs) (Sinkora and Butler, 2009, Talker et al., 2013). In the current study, PBMCs isolated at 6, 21 and 28 DPI were restimulated with WT virus and immunostained cells were analyzed by flow cytometry to identify the important T cell subsets. This analysis showed that the frequency of T-helper cells in PBMCs from vKO1-infected pigs was significantly increased by DPI 28 compared to that from mock-infected pigs (Fig. 8A), while the frequencies of cytotoxic T cells were significantly increased in PBMCs from both vKO1- and vKO2-infected pigs compared to that from mock-infected animals (Fig. 8B). These data indicate that the NK cells and the two important T cell subsets were activated, particularly in vKO1/vKO2-infected pigs, suggesting that early activation of innate immune responses following infection with nsp2TF/nsp2N-deficient mutants could enhance the innate and cell-mediated adaptive immunity, in comparison to an infection with WT virus.

## 3 Discussion

PRRSV infection is known to elicit poor innate immune responses (Albina et al., 1998, Buddaert et al., 1998, Luo et al., 2008, Miller et al., 2004, Van Reeth et al., 1999). Previous studies identified the nsp2 PLP2/DUB domain as a major innate immune antagonist (Frias-Staheli et al., 2007, Sun et al., 2010, Sun et al., 2012, van Kasteren et al., 2012). The recently identified nsp2TF and nsp2N proteins (Fang et al., 2012, Li et al., 2014) share their N-terminal ~850 amino acid residues with nsp2, including the PLP2 domain, which raised the question whether all three nsp2 variants are individually able to antagonize the innate immune response in infected cells. Potentially, they could exhibit different substrate specificities, a possibility that was highlighted by the previous observation that nsp2 and nsp2TF, which include different predicted transmembrane domains, appear to localize to different intracellular membrane structures (Fang et al., 2012), whereas the truncated nsp2N is predicted to be a cytosolic protein.

In this study, we compared the ability of full-length nsp2, nsp2TF and nsp2N to suppress the host innate immune response. These proteins were initially analyzed in an *in vitro* expression system and the results suggested that nsp2TF and nsp2N expression results in a much stronger inhibitory effect on type I IFN production. The deubiquitylation and deISGylation assays consistently showed that all three proteins have the ability to interfere with these processes which act on host cell (or viral) substrates, while nsp2N showed the strongest inhibitory effects. Interestingly, mutations targeting the catalytic residues of the PLP2 domain impaired the DUB and deISGylation activities of nsp2TF and nsp2N, but did not completely impair its immune suppression function, as demonstrated in the luciferase reporter assay (Fig. 1C). The data suggest that sequences downstream of the PLP2 domain of nsp2TF and nsp2N are also capable of suppressing innate immune functions (Fig. 1C). This may explain why in all assays nsp2N expression interfered most strongly with innate immune signaling. The activity of this part of nsp2N may depend on the localization, conformation or interactions of the protein, as both nsp2 and nsp2TF also contain this domain, but do not appear to counter innate immunity at the same level.

Obviously, we cannot formally exclude the possibility that the properties and/or relative activities of the three nsp2 variants may be different in virus-infected cells, which is why we proceeded to carefully compare the impact on the innate and cell-mediated immune (CMI) response of infection with WT virus and mutants vKO1 and vKO2, which lack expression of functional nsp2TF and nsp2TF/nsp2N, respectively. The results obtained were consistent with those from the *in vitro* expression systems, with both mutants showing a clearly impaired ability to suppress innate immune gene expression. DEG profiles identified specific biological pathways that were consistently upregulated in vKO1- or vKO2-infected cells. Most of these pathways are involved in host innate immune responses, including RIG-I-like receptor signaling pathway, Jak-STAT signaling pathway, and NF-κB signaling pathway. These data are also consistent with previous reports that PLP2 has the ability to interfere with the ubiquitination of RIG-I and IκB, thus suppressing RIG-I-mediated innate immune signaling, including NF-κB activation and downstream effects on the Jak-STAT pathway towards ISG expression (Sun et al., 2010, Sun et al., 2012, van Kasteren et al., 2012). Since the nCounter assay was originally designed for analyzing human gene expression, while our DEG profiling was conducted using PRRSV-infected MARC-145 cells (a cell line of African green monkey origin), we further confirmed the DEG profiling results using cytokine qRT-PCR to detect innate immune genes expression in PRRSV-infected MARC-145 cells as well as in PRRSV-infected swine macrophages. The results consistently showed that, compared to infection with WT virus, type I IFN (IFA 1/13, IFNB1), type III IFN (IL28B), and ISGs (IRF7, IFIH1 and IFITM1) are strongly upregulated in both MARC-145 cells and swine macrophages infected with the vKO1 and vKO2 mutants, which further implicates nsp2TF/nsp2N in innate immune suppression.

In both vKO1- and vKO2-infected cells, the nsp2 expression was not affected. The data from the *in vitro* expression system and virus infection condition made us speculate that nsp2TF and/or nsp2N might have evolved different mechanism(s) from nsp2 in the suppression of the host innate immune response. In PRRSV-infected cells, nsp2TF was found to be targeted to a different location from full-length nsp2 (Fang et al., 2012), *i.e.* the membranes of the exocytic pathway rather than the replication structures formed from modified ER membranes, a process in which nsp2 is one of the key players (Knoops et al., 2012, Oudshoorn et al., 2016). It is also worth noting that, while the TF region of nsp2TF contains a predicted TM domain, nsp2N does not contain any predicted TM domain, meaning it could be a cytosolic protein possessing unique function(s). This is also supported by the observed activity in suppression of innate immune signaling exerted by the nsp2N part downstream of PLP2, which is apparently not related to DUB activity. The mechanistic aspects of the role of PRRSV nsp2TF and nsp2N in counteracting host innate immune response need to be further studied.

Besides the panel of innate immune cytokine genes showing significant upregulation in vKO1- and vKO2-infected cells, DEG profiling also identified other upregulated genes which are associated with cellular signaling pathways. Some of these are linked to pathways of the CMI response. It is worth noting here that the expression of the DEGs associated with the antigen processing and presentation pathway was also upregulated in vKO1- and vKO2-infected cells, more specifically the expression of the human leukocyte antigen (HLA-B), which is related to major histocompatibility complex (MHC) class I (Table S1). In comparison to mock-infected cells, a 13% decrease (statistically significant) of HLA-B expression was observed in WT virus-infected cells; in contrast, expression of HLA-B was 3- and 2.5-fold increased in cells infected with vKO1 and vKO2, respectively. Since the KO1 mutations result in the expression of a C-terminally truncated form of nsp2TF, without affecting nsp2 and nsp2N expression, the data suggest that nsp2TF could be involved in modulating the expression of HLA-B. The HLA complex encodes the MHC proteins, which are expressed on the cell surface for the regulation of the human immune system (Janeway et al., 2001). In pigs, the corresponding gene complex is termed swine leukocyte antigen (SLA-1). The HLAs/SLAs present peptides on the cell surface, which are derived from proteins (both native and foreign) produced inside the cell. Upon virus infection, the HLA/SLA system related to MHC class I presents viral peptides on the cell surface so that infected cells can be recognized and destroyed by CD8+ cytotoxic T cells (Bhardwaj et al., 1994, Janeway et al., 2001). Previous studies showed that PRRSV infection down-regulates the expression of SLA class I (SLA-1) in macrophages and dendritic cells (Du et al., 2015, Park et al., 2008, Wang et al., 2007). More importantly, a recent study demonstrated that SLA-1 expression was down-regulated by the expression of nsp2TF, and the C-terminal 68 amino acids of the TF domain were concluded to be critical for this activity (Cao et al., 2016). This result is consistent with our findings presented here and further implicates nsp2TF in the CMI response during PRRSV infection. This also provides an insight into the possible molecular mechanism of impaired CMI response in PRRSV infection. The involvement of nsp2TF in modulation of the CMI response is currently under investigation in our laboratory.

We further used a nursery pig model to assess the reduced ability of nsp2TF/nsp2N-deficient mutants to interfere with the innate immune response *in vivo*. Both mutants were found to have attenuated growth in pigs and no clinical symptoms or adverse side effects of vKO1/2 infection were observed (data not shown). Viable mutant viruses could be isolated from the serum of infected pigs by 6 days post infection, demonstrating viral replication in pigs. Genome sequencing confirmed the stability of the mutations introduced in the nsp2 region of the vKO1/2 mutants. Compared to WT virus, both mutants produced lower viral loads, with mutant vKO2 consistently being most impaired. In general, IFN-α production in pigs correlated well with viral loads. In vKO1 and vKO2 infected pigs, IFN-α reached peak titer one day earlier than that in WT virus infected pigs. The stronger IFN-α response also correlated well with NK cell-mediated cytotoxicity. In our nCounter analysis, the NK cell cytotoxicity pathway was enriched in vKO1 and vKO2 infected cells. At 6 DPI, increased NK cell cytotoxicity was observed in vKO1 and vKO2-infected pigs. In addition, such a positive correlation is further demonstrated by the results of increased activation of Th1 CMI responses in vKO1 and vKO2-infected pigs. Collectively, these data suggest that expression of nsp2TF and nsp2N helps to delay the onset of the innate immune response following PRRSV infection in pigs, which may contribute to the weak induction of cell-mediated immunity during the later stage of viral infection.

It was expected that the mutations introduced in vKO1 might have less of an effect on the ability of the virus to suppress host innate immune responses, since they only C-terminally truncated nsp2TF without preventing frameshifting and the expression of nsp2N (and a truncated form of nsp2TF). Using our *in vitro* gene expression assays (Fig. 1C), both nsp2TF and nsp2N were found to antagonize innate immune signaling, with nsp2N expression having the strongest effect. The vKO2 mutations knock out the expression of both nsp2TF and nsp2N, which could have been expected to exert a stronger effect. However, when tested in the context of PRRSV infection, the qRT-PCR results consistently showed higher cytokine gene expression levels in vKO1-infected cells, although the difference was not statistically significant. Moreover, vKO1-infected pigs showed higher frequencies of T-helper and cytotoxic T cells than vKO2-infected pigs (Fig. 8). One explanation for this phenomenon could be the reduced ability of mutant vKO2 to replicate in infected cells and animals compared to vKO1. Even if vKO1 has a lower intrinsic IFN-inducing potential than vKO2, this could be compensated by a higher level of viral replication, eventually resulting in a comparable or stronger stimulation of the immune responses in infected cells or animals.

In conclusion, our results show that nsp2TF and nsp2N can modulate the host's innate immune responses against infection with PRRSV and, most likely, other arteriviruses. Recombinant viruses with impaired expression of these frameshift-derived nsp2 variants are attenuated upon infection of animals. Since the ribosomal frameshift site in the nsp2-coding region is highly conserved among different PRRSV strains, manipulating the expression of nsp2TF and nsp2N may provide a rational basis for developing improved PRRSV vaccines in the future.

## 4 Materials and methods

### 4.1 Viruses and cells

PRRSV-2 isolate SD95-21 (GenBank accession no. KC469618) and its nsp2TF/nsp2N-deficient mutants were used in all experiments. BHK-21 cells were used for initial transfection and recovery of recombinant viruses. MARC-145 cells were used for recombinant virus production and subsequent experiments. These cells were maintained in minimum essential medium (Invitrogen) supplemented with 10% heat-inactivated fetal bovine serum and antibiotics (100 units/mL of penicillin, 100 µg/mL of streptomycin and 0.25 µg/mL of fungizone) at 37 °C with 5% CO_2_. Porcine alveolar macrophages (PAMs) were obtained by lung lavage of 6-week-old PRRSV-naive piglets using a method described previously (Zeman et al., 1993). The Sendai virus (SeV) Cantell strain was grown in embryonated chicken eggs, and virus titer was determined by hemagglutination assay using chicken red blood cells as described previously (Yonemitsu and Kaneda, 1999).

### 4.2 Plasmids

As indicated in Fig. 1A, a panel of plasmids expressing different regions of nsp2 was constructed by cloning corresponding viral genomic sequences into plasmid vector p3xFLAG-*Myc*-CMV-24 (Sigma-Aldrich, St. Louis, MO) using the restriction sites of *Not*I and *Bam*HI. This panel of plasmids was designated as pFLAG-nsp2, pFLAG-nsp2TF, pFLAG-nsp2N, pFLAG-PLP2, pFLAG-nsp2_(599–1233)_, pFLAG-nsp2TF_(599–1402)_ and pFLAG-nsp2_(599–1579)._ In the nsp2TF construct, the -2/-1 PRF region was modified to ensure expression of only nsp2TF using QuickChange™ site-directed mutagenesis kit (Agilent Technologies, Santa Clara, CA). Based on these constructs, a panel of mutant plasmids expressing nsp2-related proteins was created using QuickChange™ site-directed mutagenesis kit (Agilent Technologies, Santa Clara, CA), in which PLP2 protease function was inactivated by introducing alanine substitutions at Cys^433^ and His^503^ (C/H>A). This panel of plasmids was designated as pFLAG-nsp2-C/H>A, pFLAG-nsp2TF-C/H>A, pFLAG-nsp2N-C/H>A, or pFLAG-PLP2-C/H>A. The full-length cDNA clones, pCMV-SD95-21 and pCMV-SD95-21-KO2, were used to recover SD95-21 WT virus and mutant vKO2 as we described previously (Li et al., 2014). To create a full-length cDNA clone in which the TF ORF was truncated, three stop codons were introduced using QuickChange™ site-directed mutagenesis kit to generate infectious clone of pCMV-SD95-21-KO1 (Fig. 3A) (Fang et al., 2012). In luciferase reporter assay, two reporter plasmids, the p125-Luc and pRL-SV40 were used as described previously (Sun et al., 2010). The p125-Luc reporter plasmid was kindly provided by Takashi Fujita (Yoneyama et al., 1996) and expresses the firefly luciferase under the control of the IFN-β promoter. The pRL-SV40 plasmid that expresses a *Renilla* luciferase under the control of a simian virus 40 (SV40) promoter was purchased from Promega (Madison, WI).

### 4.3 Antibodies

To detect the expression of nsp2, nsp2TF, nps2N and PLP2, the α-FLAG monoclonal antibody (mAb) M2 (Sigma-Aldrich, St. Louis, MO) and mAb 140-68 specific for the PRRSV-2 PLP2 domain were used. A polyclonal antibody (pAb) specifically recognizing the unique C-terminal domain of nsp2TF was used to detect full-length nsp2TF (Li et al., 2014). A rabbit pAb specifically recognizing the nsp2 C‐terminus was used to detect full-length nsp2 (Guo et al., 2016). The mAb SDOW17 that is specific for the PRRSV nucleocapsid protein was used to detect viral infection in cell culture (Nelson et al., 1993). The anti-GAPDH pAb sc-25778 (Santa Cruz Biotechnology, Dallas, TX) was used to detect the expression of housekeeping gene GAPDH.

### 4.4 Luciferase reporter assay

HEK-293T cells were transfected with 0.5 µg plasmid DNA of p125-luc (a luciferase reporter plasmid containing IFN-β promoter), 20 ng plasmid DNA of pRL-SV40, and 1.0 µg plasmid DNA expressing 3xFLAG-tagged nsp2, nsp2TF, nsp2N, nsp2_(599–1233)_, nsp2TF_(599–1402)_, nsp2_(599–1579)_, PLP2, nsp2-C/H>A, nsp2TF-C/H>A, nsp2N-C/H>A, PLP2-C/H>A, or empty vector (EV). Transfection was conducted using TransIT®-LT1 Transfection Reagent (Mirus Bio LLC, Madison, WI) following the manufacturer's instructions. At 24 h post transfection, cells were stimulated by infection with SeV at 100 HA unit per mL for 16 h. Cells were harvested and subjected to a reporter gene assay using a dual luciferase reporter system (Promega, Madison, WI) according to the manufacturer's instructions. Firefly or Renilla luciferase activity was measured in FLUOstar Omega (BMG Labtech). Relative luciferase activities were calculated by normalizing the firefly luciferase to Renilla luciferase activities. Cell lysates were further used on SDS-PAGE and Western blot analysis to evaluate protein expression. To determine the possible cytotoxicity caused by transfection and protein expression, triplicate cell cultures were subjected to test cell viability using CellTiter 96® AQueous One Solution Cell Proliferation Assay (MTS) (Promega, Madison, WI), following the manufacturer's instructions.

### 4.5 Radioimmunoprecipitation

MARC-145 cells were infected with PRRSV WT or nsp2TF/nsp2N-deficient mutants at a multiplicity of infection (MOI) of 0.1 for 24 h. After three washes with PBS, cells starved for 30 min in methionine- and cysteine-free medium (Thermo Fisher Scientific, Waltham, MA). Subsequently, protein synthesis in the infected cells was metabolically labeled for 2 h in methionine- and cysteine-free medium supplemented with 200 mCi [^35^S]methionine/cysteine mixture (Perkin-Elmer). After labelling, cells were harvested in lysis buffer [20 mM Tris/HCl (pH 7.6), 150 mM NaCl, 1% NP-40, 0.1% sodium deoxycholate, 0.1% SDS] and cell debris were removed by centrifugation. Immunoprecipitaion was performed with mAb 140-68 that recognizes PLP2 domain of nsp2, nsp2TF and nsp2N as described previously (Li et al., 2012). The protein complexes were dissolved in Laemmli sample buffer and heated at 96 °C for 6 min prior to loading onto Novex™ 6% Tris-Glycine Mini Gel (Thermofisher scientific, Waltham, MA). Gel was dried and exposed to autoradiography film (Sigma-Aldrich, St. Louis, MO).

### 4.6 Western blot analysis

To evaluate protein expression in DNA-transfected or virus-infected cells, western blot analysis was performed using the method described previously (Li et al., 2016, Li et al., 2013). Briefly, cells were harvested with passive lysis buffer of Dual-Luciferase® Reporter Assay System (Promega, Madison, WI) or Pierce IP Lysis Buffer (Thermo Fisher Scientific, Waltham, MA) containing protease inhibitor cocktail (Sigma-Aldrich, St. Louis, MO). After being clarified by centrifugation at 15,000*g* for 15 min, cell lysates were mixed with Laemmli sample buffer (4X) and heated at 95 °C for 6 min or 37 °C for 30 min. Proteins were separated by sodium dodecyl sulfate-polyacrylamide gel electrophoresis (SDS-PAGE) and transferred onto a nitrocellulose membrane. After overnight blocking with 5% skim milk at 4 °C, the membrane was incubated with the primary antibody at an appropriate dilution at room temperature for 1 h. The membrane was washed three times with PBS containing 0.05% TWEEN 20 (PBST), and then incubated with the secondary antibody, IRDye® 800CW Goat anti-Mouse IgG (H + L) or/and IRDye® 680RD Goat anti- Rabbit IgG (H + L) (LI-COR Biosciences, Lincoln, NE) at an appropriate dilution for 1 h at room temperature. After extensively washing with PBST, the target proteins were visualized using a digital image system (Odyssey infrared imaging system; LI-COR Biosciences, Lincoln, NE).

### 4.7 Recovery of recombinant viruses from infectious cDNA clones

The recombinant viruses were launched by transfecting BHK-21 cells as described previously (Li et al., 2013). Briefly, BHK-21 cells (70–80% confluency) were transfected with 2 μg of the type 2 PRRSV full-length cDNA clone of pCMV-SD95-21 or the mutated full-length cDNA clones (pCMV-SD95-21-KO1 and pCMV-SD95-21-KO2) using FuGENE HD reagent (Promega, Madison, WI). At 48 h post transfection, cell culture supernatant was harvested and passaged onto MARC-145 cells. The viability of recombinant viruses was confirmed by indirect immunofluorescence assay using mAb SDOW17. The recombinant viruses were serially passaged on MARC-145 cells, and the passage 4 viruses were used for further analysis.

### 4.8 In vitro growth characterization of recombinant viruses in cell culture

Growth kinetics of the recombinant and parental viruses were examined by infecting MARC-145 cells at an MOI of 0.01. Infected cells were collected at 0, 12, 24, 36, 48, 60, 72 h post-infection (hpi). Viral titers were determined by microtitration assay on MARC-145 cells and calculated as TCID_50_/mL according to the Reed and Muench method (Reed and Muench, 1938).

### 4.9 nCounter mRNA profiling for detecting immune gene expression

MARC-145 cells were infected with WT PRRSV SD95-21, vKO1, or vKO2 at an MOI of 1.0. At 12 hpi, cells were harvested and subjected to total RNA extraction using SV Total RNA Isolation kit (Promega, Madison, WI) following the manufacturer's instructions. Total RNA (100 ng/sample) was used in cellular gene expression profiling. The expression levels of 579 immunological genes were evaluated by the nCounter assay with nCounter® Human Immunology v2 kit according to the manufacturer's instruction (NanoString Technologies, Seatle, WA), and 15 housekeeping genes were included for data normalization. Briefly, hybridization reactions were prepared with 5 μl diluted sample RNA according to the manufacturer's instruction. After 18 h of hybridization at 65 °C, the excess probes were removed and the hybridized probe/target complexes were immobilized in an nCounter cartridge using the nCounter Prep Station (Nanostring Technologies, Seatle, WA). Sample Cartridges were placed in a Digital Analyzer for data collection. The gene expression data were analyzed with nSolver 2.6 according to the manufacturer's instruction (NanoString Technologies, Seatle, WA). Using gene expression in uninfected control cells as a reference, genes in virus-infected cells with a p-value lower than 0.05 and a fold change greater than 2 were defined as differentially expressed genes (DEGs).

### 4.10 KEGG pathway enrichment and protein-protein interaction network analysis

The Database for Annotation, Visualization and Integrated Discovery (DAVID) (Huang et al., 2009a, Huang et al., 2009b) was used to perform KEGG functional enrichment analysis of biological pathways that significantly enriched with DEGs from individual viral infection. A minimum count of 4 genes was used as the cut-off for determination of enriched biological pathways, and the p-values adjusted by Benjamini-Hochberg correction less than 0.05 were defined as significant enrichment. Protein-protein interaction (PPI) networks were constructed using Search Tool for the Retrieval of Interacting Genes/Proteins (STRING v10.0, http://string-db.org) (Szklarczyk et al., 2015) using DEG list from each viral infection. PPI confidence networks were generated using the confidence view option at a medium confidence of 0.400.

### 4.11 Quantitative RT-PCR for immune gene detection

To verify the results of nCounter gene expression profiling, the expression levels of selected genes were further evaluated by quantitative RT-PCR using the total RNA of the same sample for nCounter analysis. Briefly, cDNA was generated with 1 μg total RNA using SuperScript VILO cDNA Synthesis Kit (Life Technologies, Carlsbad, CA). According to the manufacturer's instruction, PCR reaction was formulated with 10 μl TaqMan Fast Advanced Master Mix (Applied Biosystems, Foster City, CA), 1 μl cDNA, and 1 μl of predesigned primer/probe sets (Applied Biosystems, Foster City, CA) for IFN-α, IFN-β, IRF-7, IL-28B, IFIT1, IFITM1 and TBP (TATA Box Binding Protein). Reactions were completed on CFX96 Real-Time PCR system (Bio-Rad) under the following conditions: 2 min of 50 °C for UNG activation, 20 s of 95 °C for polymerase activation, 40 amplification cycles of 30 s of 95 °C and 30 s of 60 °C. The mRNA expression levels of IFN-α, IFN-β, IRF-7, IL-28B, IFIT1 and IFITM1 were normalized to the endogenous TBP mRNA level.

To assess the ability of mutant viruses to stimulate innate immune responses in virus-infected swine macrophages, PAMs were infected with WT virus, vKO1 or vKO2 at an MOI of 1.0. At 12 hpi, cells were harvested in TRIzol LS (Life Technologies, Carlsbad, CA) and total cellular RNA was extracted according to the manufacturer's instruction. The mRNA expression levels of IFN-α, IFN-β, IRF-7, IL-28B, IFIT1, IFITM1 were quantified by quantitative RT-PCR using predesigned probe/primer sets (Applied Biosystems, Foster City, CA) and normalized to the housekeeping gene GAPDH mRNA.

### 4.12 Pig groups, sample collection and preparation

A total of thirty-six 4-week-old PRRSV-naive pigs were obtained from a certified PRRSV-negative herd. They were divided randomly into 4 groups (n=9) and housed separately in an animal isolation facility. After a four-day acclimation period, group 1 pigs were mock-infected with cell culture medium, while pigs from group 2–4 were inoculated with WT virus (group 2), vKO1 (group 3) or vKO2 (group 4). Pigs were immunized through both intranasal (IN) and intramuscular (IM) routes with 1 mL (1×10^6^ TCID_50_) of the virus suspension in MEM to each nostril and to each side of the neck. Pigs were observed daily and serum samples were collected at 0, 1, 2, 6, 21, 28 DPI. Three pigs from each group were sequentially euthanized at 6, 21, and 28 DPI. During necropsy, whole blood was collected from each pig for preparation of peripheral blood mononuclear cells (PBMCs), and gross lung lesions were evaluated using the method described previously (Halbur et al., 1995). The pig experiment was performed according to the protocol approved by the Institutional Animal Care and Use Committee (IACUC) of The Ohio State University, Ohio.

### 4.13 Quantification of viral load

For the detection of viral RNA and quantification of viral load, serum samples were examined using a quantitative RT-PCR method as described previously (Li et al., 2016).

### 4.14 Analysis of swine cytokine response

Serum samples collected at 0, 1, 2 and 6 DPI were used to evaluate IFN-α production using ProcartaPlex Porcine IFN alpha Simplex kit (eBioscience, San Diego, CA) per manufacturer's instructions.

### 4.15 Pig NK cell cytotoxic assay

Pig natural killer (NK) cell-mediated cytotoxicity was determined with an immunofluorescence-based assay using a modified method described previously (Lecoeur et al., 2001). A 7-aminoactinomycin D (7-AAD)/carboxyfluorescein succinimidyl ester (CFSE) cell-mediated cytotoxicity assay kit (Cayman Chemical, Ann Arbor, MI) was used. PBMCs were used as the source of NK cells (effectors), and K562 (human myeloblastoid cell line) cells were used as target cells.

### 4.16 Flow cytometry analysis

To measure the frequencies of virus-specific lymphocyte population, PBMCs isolated at 6, 21 and 28 DPI were restimulated with WT virus at an MOI of 0.1. At 72-h post stimulation, cells were subjected to flow cytometry analysis to determine the frequency of T-helper cells (CD3^+^CD4^+^CD8α^-^) and cytotoxic T cells (CD3^+^CD4^-^CD8αβ^+^) using the modified method described previously (Binjawadagi et al., 2014, Dhakal et al., 2017). Briefly, PBMCs plated in a 96 well-plate were surface-labeled with swine lymphocyte specific fluorochrome- or biotin-conjugated monoclonal antibody, and then stained with fluorochrome-labeled anti-mouse isotype specific antibody or streptavidin. Antibodies used in the flow cytometry were anti-porcine CD3, CD4α and CD8α (Southernbiotech, AL) and CD8β (BD Biosciences, CA). Immunostained cells were acquired using the FACS Aria II (BD Biosciences) flow cytometer and analyzed using FlowJo (Tree Star, Ashland, OR, USA) software. All specific cell population frequencies were presented as the percentage of CD3^+^ cells in PBMCs.

### 4.17 Statistical analysis

Statistical analysis was performed using GraphPad InStat version 5.0 (GraphPad Software). Comparisons among treatment groups were performed using one-way analysis of variance (ANOVA) followed by Tukey's post hoc test to determine the statistical significance. A p-value below 0.05 was considered to indicate a statistically significant difference between treatment groups. Due to co-housing of pigs infected with the same virus (WT or a mutant virus), co-housed pigs are not strictly speaking completely independent samples, thus p-values in Fig. 6, Fig. 7, Fig. 8 should be treated with caution.
