# Supplementary material for: Nonstructural proteins nsp2TF and nsp2N of porcine reproductive and respiratory syndrome virus (PRRSV) play important roles in suppressing host innate immune responses
Source: Virology. 2018 Apr;517:164–76. doi: 10.1016/j.virol.2017.12.017 (PMC5884420; doi:10.1016/j.virol.2017.12.017)
Supplement: Supplementary file 1 — Supplementary material [file mmc4.docx]

**Table S1.** DEGs identified in Marc-145 cell infected with wild type and mutant viruses

| **WT virus versus Mock** | | | **vKO1 versus Mock** | | |
| --- | --- | --- | --- | --- | --- |
| Gene Name | Accession # | Fold change | Gene Name | Accession # | Fold change |
| C9 | NM_001737.3 | 3.12 | BST2 | NM_004335.2 | 10.77 |
| CCL2 | NM_002982.3 | 3.81 | C4A/B | NM_007293.2 | 3.03 |
| CCL20 | NM_004591.1 | 2.05 | C9 | NM_001737.3 | 6.72 |
| CDH5 | NM_001795.3 | 2.01 | CASP1 | NM_001223.3 | 5.56 |
| CSF2 | NM_000758.2 | 2.52 | CASP8 | NM_001228.4 | 2.52 |
| IL8 | NM_000584.2 | 2.25 | CCL2 | NM_002982.3 | 13.33 |
| PRDM1 | NM_001198.3 | 2.67 | CCL20 | NM_004591.1 | 4.85 |
| RAG1 | NM_000448.2 | 2.55 | CCL4 | NM_002984.2 | 5.05 |
| TNFAIP3 | NM_006290.2 | 2.22 | CCL5 | NM_002985.2 | 181.39 |
| TRAF1 | NM_005658.3 | 2.61 | CCL7 | NM_006273.2 | 2.86 |
| LILRA1 | NM_006863.1 | -2.24 | CCRL1 | NM_016557.2 | 11.74 |
| **vKO2 versus Mock** | | | CD1D | NM_001766.3 | 2.16 |
| Gene Name | Accession # | Fold change | CD274 | NM_014143.3 | 49.82 |
| BST2 | NM_004335.2 | 9.02 | CD34 | NM_001025109.1 | 2.38 |
| C4A/B | NM_007293.2 | 2.45 | CD45R0 | NM_080921.3 | 2.19 |
| C9 | NM_001737.3 | 10.91 | CEACAM1 | NM_001712.3 | 3.09 |
| CASP1 | NM_001223.3 | 3.43 | CISH | NM_145071.2 | 2.14 |
| CCL2 | NM_002982.3 | 12.62 | CLEC7A | NM_197954.2 | 3.02 |
| CCL20 | NM_004591.1 | 7.02 | CSF1 | NM_000757.4 | 2.84 |
| CCL4 | NM_002984.2 | 4.6 | CSF2 | NM_000758.2 | 2.11 |
| CCL5 | NM_002985.2 | 107.7 | CTSS | NM_004079.3 | 2.44 |
| CCL7 | NM_006273.2 | 2.03 | CX3CL1 | NM_002996.3 | 2.16 |
| CCRL1 | NM_016557.2 | 7.35 | CXCL10 | NM_001565.1 | 208.13 |
| CD274 | NM_014143.3 | 34.99 | CXCL11 | NM_005409.4 | 2463.04 |
| CD34 | NM_001025109.1 | 2.4 | CXCL2 | NM_002089.3 | 4.03 |
| CISH | NM_145071.2 | 2.99 | CXCL9 | NM_002416.1 | 96.18 |
| CSF1 | NM_000757.4 | 2.68 | CXCR4 | NM_003467.2 | 2.65 |
| CSF2 | NM_000758.2 | 3.06 | EGR1 | NM_001964.2 | 6.19 |
| CXCL10 | NM_001565.1 | 129.88 | EGR2 | NM_000399.3 | 2.01 |
| CXCL11 | NM_005409.4 | 1386.08 | FADD | NM_003824.2 | 3.05 |
| CXCL2 | NM_002089.3 | 5.44 | FAS | NM_000043.3 | 2.07 |
| CXCL9 | NM_002416.1 | 59.82 | GBP1 | NM_002053.1 | 119.81 |
| CXCR4 | NM_003467.2 | 2.22 | HLA-B | NM_005514.6 | 2.98 |
| EGR1 | NM_001964.2 | 4.73 | HLA-DOB | NM_002120.3 | 2.73 |
| GBP1 | NM_002053.1 | 71.36 | HLA-DQA1 | NM_002122.3 | 2.05 |
| HLA-B | NM_005514.6 | 2.47 | ICAM1 | NM_000201.2 | 2.34 |
| HLA-DOB | NM_002120.3 | 2.21 | IDO1 | NM_002164.3 | 130.51 |
| ICAM1 | NM_000201.2 | 3.16 | IFI16 | NM_005531.1 | 6.23 |
| IDO1 | NM_002164.3 | 71.25 | IFI35 | NM_005533.3 | 5.9 |
| IFI16 | NM_005531.1 | 4.88 | IFIH1 | NM_022168.2 | 49.88 |
| IFI35 | NM_005533.3 | 4.77 | IFIT2 | NM_001547.4 | 104.49 |
| IFIH1 | NM_022168.2 | 38.96 | IFITM1 | NM_003641.3 | 8.64 |
| IFIT2 | NM_001547.4 | 71.94 | IFNA1/13 | NM_024013.1 | 20.61 |
| IFITM1 | NM_003641.3 | 6.71 | IFNB1 | NM_002176.2 | 267.51 |
| IFNA1/13 | NM_024013.1 | 8.76 | IL12A | NM_000882.2 | 5.04 |
| IFNB1 | NM_002176.2 | 225.19 | IL15 | NM_172174.1 | 2.45 |
| IL12A | NM_000882.2 | 3.84 | IL17F | NM_052872.3 | 2.37 |
| IL15 | NM_172174.1 | 2.61 | IL18RAP | NM_003853.2 | 2.09 |
| IL17F | NM_052872.3 | 3.17 | IL19 | NM_013371.3 | 3 |
| IL1A | NM_000575.3 | 5.76 | IL1A | NM_000575.3 | 6.57 |
| IL1RL2 | NM_003854.2 | 2.59 | IL1B | NM_000576.2 | 2.73 |
| IL28A | NM_172138.1 | 76.18 | IL1RL2 | NM_003854.2 | 2.6 |
| IL28A/B | NM_172139.2 | 29.07 | IL28A | NM_172138.1 | 96.73 |
| IL29 | NM_172140.1 | 146.44 | IL28A/B | NM_172139.2 | 35.66 |
| IL6 | NM_000600.1 | 4.57 | IL29 | NM_172140.1 | 201.87 |
| IL8 | NM_000584.2 | 4.56 | IL6 | NM_000600.1 | 3.97 |
| IRF1 | NM_002198.1 | 17.11 | IL8 | NM_000584.2 | 3.64 |
| IRF7 | NM_001572.3 | 26.24 | IRF1 | NM_002198.1 | 19.58 |
| IRF8 | NM_002163.2 | 17.38 | IRF4 | NM_002460.1 | 2.01 |
| LCP2 | NM_005565.3 | 2.24 | IRF7 | NM_001572.3 | 30.97 |
| MR1 | NM_001531.2 | 2.95 | IRF8 | NM_002163.2 | 28.54 |
| MYD88 | NM_002468.3 | 4.86 | LCP2 | NM_005565.3 | 3.5 |
| NFIL3 | NM_005384.2 | 2.02 | LIF | NM_002309.3 | 2.04 |
| NFKBIA | NM_020529.1 | 3.1 | LY96 | NM_015364.2 | 2.32 |
| NFKBIZ | NM_001005474.1 | 2.28 | MRC1 | NM_002438.2 | 2.89 |
| NOD2 | NM_022162.1 | 3.03 | MX1 | NM_002462.2 | 3.2 |
| PLAUR | NM_001005376.1 | 4.53 | MYD88 | NM_002468.3 | 6.33 |
| PML | NM_002675.3 | 5.15 | NFIL3 | NM_005384.2 | 2.24 |
| PRDM1 | NM_001198.3 | 5.82 | NFKBIA | NM_020529.1 | 3.04 |
| PSMB10 | NM_002801.2 | 5.04 | NOD2 | NM_022162.1 | 3.27 |
| PSMB8 | NM_004159.4 | 14.68 | NOS2 | NM_000625.4 | 2.16 |
| PSMB9 | NM_002800.4 | 21.04 | PLAUR | NM_001005376.1 | 5.95 |
| RAG1 | NM_000448.2 | 5.88 | PML | NM_002675.3 | 5.05 |
| RARRES3 | NM_004585.3 | 2.72 | PRDM1 | NM_001198.3 | 4.96 |
| SELE | NM_000450.2 | 6.12 | PSMB10 | NM_002801.2 | 7.43 |
| SOCS1 | NM_003745.1 | 11.36 | PSMB8 | NM_004159.4 | 23.21 |
| SOCS3 | NM_003955.3 | 2.38 | PSMB9 | NM_002800.4 | 31.43 |
| STAT2 | NM_005419.2 | 6.26 | RAG1 | NM_000448.2 | 3.49 |
| TAP1 | NM_000593.5 | 13.57 | RARRES3 | NM_004585.3 | 4.24 |
| TAP2 | NM_000544.3 | 4.32 | SELE | NM_000450.2 | 3.35 |
| TICAM1 | NM_014261.1 | 2.19 | SOCS1 | NM_003745.1 | 15.46 |
| TLR3 | NM_003265.2 | 3.56 | SOCS3 | NM_003955.3 | 2.05 |
| TNF | NM_000594.2 | 2.73 | STAT2 | NM_005419.2 | 7.75 |
| TNFAIP3 | NM_006290.2 | 6.21 | TAP1 | NM_000593.5 | 17.78 |
| TNFAIP6 | NM_007115.2 | 2.02 | TAP2 | NM_000544.3 | 5.66 |
| TNFSF10 | NM_003810.2 | 5.75 | TLR2 | NM_003264.3 | 2.06 |
| TNFSF13B | NM_006573.4 | 4.3 | TLR3 | NM_003265.2 | 5.26 |
| TRAF1 | NM_005658.3 | 7.22 | TNF | NM_000594.2 | 2.78 |
| CTLA4_all | NM_005214.3 | -2.1 | TNFAIP3 | NM_006290.2 | 4.93 |
| ITLN1 | NM_017625.2 | -2.52 | TNFAIP6 | NM_007115.2 | 2.36 |
| ZAP70 | NM_001079.3 | -2.08 | TNFSF10 | NM_003810.2 | 8.8 |
|  |  |  | TNFSF13B | NM_006573.4 | 8.54 |
|  |  |  | TNFSF8 | NM_001244.3 | 2.2 |
|  |  |  | TRAF1 | NM_005658.3 | 8.67 |
|  |  |  | IL23A | NM_016584.2 | -2.01 |
|  |  |  | TGFBR1 | NM_004612.2 | -2.12 |

**Table S2.** KEGG pathway enrichment from differentially expressed genes induced by viral infection

| **WT virus** | | | | | |
| --- | --- | --- | --- | --- | --- |
| Term | Genes | Count | % | P-Value | Benjamini |
| hsa04668:TNF signaling pathway | TRAF1, CSF2, CCL2, CCL20, TNFAIP3 | 5 | 50 | 2.03E-09 | 8.34E-08 |
| hsa04060:Cytokine-cytokine receptor interaction | CSF2, CCL2, CCL20, CXCL8 | 4 | 40 | 1.37E-05 | 1.87E-04 |
| **vKO1** | | | | | |
| Term | Genes | Count | % | P-Value | Benjamini |
| hsa04668:TNF signaling pathway | TRAF1, ICAM1, CSF2, IL6, TNF, CCL2, SOCS3, CSF1, CXCL2, NFKBIA, FADD, CX3CL1, IL15, CCL5, CXCL10, LIF, NOD2, CCL20, CASP8, IL1B, FAS, TNFAIP3, SELE | 23 | 25 | 5.55E-42 | 1.92E-40 |
| hsa04060:Cytokine-cytokine receptor interaction | CSF2, TNF, CCL2, CSF1, CXCL9, CXCL8, CX3CL1, IL15, CXCL11, CCL5, CCL4, CCL7, CXCL10, LIF, IFNA1, CCL20, CXCR4, IL1B, FAS, IL1A, IL6, IL18RAP, TNFSF8, TNFSF10, TNFSF13B, IFNB1, IL12A | 27 | 29.34783 | 9.82E-39 | 2.55E-37 |
| hsa04620:Toll-like receptor signaling pathway | IL6, TNF, LY96, CXCL9, TLR2, CXCL8, TLR3, NFKBIA, FADD, CXCL11, CCL5, CCL4, CXCL10, IFNA1, MYD88, IFNB1, IRF7, CASP8, IL12A, IL1B | 20 | 21.73913 | 3.31E-32 | 6.89E-31 |
| hsa04630:Jak-STAT signaling pathway | CSF2, IL6, SOCS3, IL19, SOCS1, IL15, CISH, STAT2, LIF, IFNA1, IFNL2, IFNB1, IL12A, IFNL3 | 14 | 15.21739 | 2.39E-19 | 2.26E-18 |
| hsa04062:Chemokine signaling pathway | CCL2, CXCL2, CXCL9, CXCL8, NFKBIA, CX3CL1, CXCL11, CCL5, CCL4, CCL7, STAT2, CXCL10, CCL20, CXCR4 | 14 | 15.21739 | 3.02E-19 | 2.62E-18 |
| hsa04621:NOD-like receptor signaling pathway | IL6, NOD2, TNF, CCL2, CASP8, CXCL8, NFKBIA, IL1B, CCL5, TNFAIP3, CASP1 | 11 | 11.95652 | 3.42E-18 | 2.54E-17 |
| hsa04622:RIG-I-like receptor signaling pathway | IFIH1, IFNA1, TNF, IFNB1, IRF7, CASP8, IL12A, CXCL8, NFKBIA, FADD, CXCL10 | 11 | 11.95652 | 3.08E-17 | 2.00E-16 |
| hsa04623:Cytosolic DNA-sensing pathway | IL6, IFNA1, IFNB1, IRF7, NFKBIA, IL1B, CCL5, CASP1, CCL4, CXCL10 | 10 | 10.86957 | 1.39E-16 | 6.66E-16 |
| hsa04064:NF-kappa B signaling pathway | TRAF1, ICAM1, TNF, MYD88, TNFSF13B, LY96, CXCL8, NFKBIA, IL1B, TNFAIP3, CCL4 | 11 | 11.95652 | 1.23E-14 | 6.11E-14 |
| hsa04650:Natural killer cell mediated cytotoxicity | CSF2, ICAM1, IFNA1, TNFSF10, TNF, IFNB1, FAS, LCP2 | 8 | 8.695652 | 5.60E-10 | 1.76E-09 |
| hsa04514:Cell adhesion molecules (CAMs) | ICAM1, PTPRC, CD34, CD274, HLA-B, SELE, HLA-DOB, HLA-DQA1 | 8 | 8.695652 | 2.25E-09 | 6.89E-09 |
| hsa04640:Hematopoietic cell lineage | CSF2, IL6, TNF, CD34, CSF1, IL1B, IL1A, CD1D | 8 | 8.695652 | 3.38E-09 | 1.01E-08 |
| hsa04612:Antigen processing and presentation | TNF, TAP2, TAP1, HLA-B, CTSS, HLA-DOB, HLA-DQA1 | 7 | 7.608696 | 8.76E-09 | 2.53E-08 |
| hsa04210:Apoptosis | TNFSF10, TNF, CASP8, NFKBIA, FADD, FAS | 6 | 6.521739 | 3.10E-08 | 8.48E-08 |
| hsa04672:Intestinal immune network for IgA production | IL6, TNFSF13B, CXCR4, IL15, HLA-DOB, HLA-DQA1 | 6 | 6.521739 | 4.24E-07 | 1.07E-06 |
| hsa04660:T cell receptor signaling pathway | CSF2, PTPRC, TNF, NFKBIA, LCP2 | 5 | 5.434783 | 2.50E-05 | 5.65E-05 |
| hsa04917:Prolactin signaling pathway | SOCS3, SOCS1, IRF1, CISH | 4 | 4.347826 | 6.91E-05 | 1.50E-04 |
| hsa04151:PI3K-Akt signaling pathway | IL6, IFNA1, IFNB1, CSF1, TLR2 | 5 | 5.434783 | 7.13E-05 | 1.51E-04 |
| hsa04010:MAPK signaling pathway | TNF, IL1B, FAS, IL1A | 4 | 4.347826 | 4.44E-04 | 8.88E-04 |
| **vKO2** | | | | | |
| Term | Genes | Count | % | P-Value | Benjamini |
| hsa04060:Cytokine-cytokine receptor interaction | CSF2, IL6, TNF, CCL2, CSF1, CXCL9, CXCL8, IL15, CXCL11, CCL5, CCL4, CCL7, CXCL10, IFNA1, TNFSF10, TNFSF13B, CCL20, CXCR4, IFNB1, IL12A, IL1A | 21 | 28.76712 | 6.85E-30 | 2.12E-28 |
| hsa04668:TNF signaling pathway | TRAF1, ICAM1, CSF2, IL6, TNF, CCL2, SOCS3, CSF1, CXCL2, NFKBIA, IL15, CCL5, CXCL10, NOD2, CCL20, TNFAIP3, SELE | 17 | 23.28767 | 7.98E-30 | 1.85E-28 |
| hsa04620:Toll-like receptor signaling pathway | IL6, TNF, CXCL9, CXCL8, NFKBIA, TLR3, CXCL11, CCL5, CCL4, CXCL10, IFNA1, MYD88, IFNB1, IRF7, TICAM1, IL12A | 16 | 21.91781 | 1.22E-25 | 2.27E-24 |
| hsa04062:Chemokine signaling pathway | CCL2, CCL20, CXCR4, CXCL2, CXCL9, CXCL8, NFKBIA, CCL5, CXCL11, CCL4, CCL7, STAT2, CXCL10 | 13 | 17.80822 | 7.46E-19 | 9.91E-18 |
| hsa04630:Jak-STAT signaling pathway | CSF2, IL6, IFNA1, IFNL2, SOCS3, IFNB1, SOCS1, IL12A, IL15, IFNL3, CISH, STAT2 | 12 | 16.43836 | 5.45E-17 | 5.07E-16 |
| hsa04623:Cytosolic DNA-sensing pathway | IL6, IFNA1, IFNB1, IRF7, NFKBIA, CCL5, CASP1, CCL4, CXCL10 | 9 | 12.32877 | 2.56E-15 | 1.98E-14 |
| hsa04621:NOD-like receptor signaling pathway | IL6, NOD2, TNF, CCL2, CXCL8, NFKBIA, CCL5, TNFAIP3, CASP1 | 9 | 12.32877 | 8.58E-15 | 6.12E-14 |
| hsa04622:RIG-I-like receptor signaling pathway | IFIH1, IFNA1, TNF, IFNB1, IRF7, IL12A, CXCL8, NFKBIA, CXCL10 | 9 | 12.32877 | 4.61E-14 | 2.86E-13 |
| hsa04064:NF-kappa B signaling pathway | TRAF1, ICAM1, TNF, MYD88, TNFSF13B, TICAM1, CXCL8, NFKBIA, TNFAIP3, CCL4 | 10 | 13.69863 | 6.49E-14 | 3.77E-13 |
| hsa04650:Natural killer cell mediated cytotoxicity | CSF2, ICAM1, IFNA1, TNFSF10, TNF, IFNB1, LCP2 | 7 | 9.589041 | 5.39E-09 | 2.09E-08 |
| hsa04514:Cell adhesion molecules (CAMs) | ICAM1, CD34, CD274, HLA-B, SELE, HLA-DOB | 6 | 8.219178 | 7.44E-07 | 2.16E-06 |
| hsa04640:Hematopoietic cell lineage | CSF2, IL6, TNF, CD34, CSF1, IL1A | 6 | 8.219178 | 9.89E-07 | 2.79E-06 |
| hsa04672:Intestinal immune network for IgA production | IL6, TNFSF13B, CXCR4, IL15, HLA-DOB | 5 | 6.849315 | 5.72E-06 | 1.40E-05 |
| hsa04612:Antigen processing and presentation | TNF, TAP2, TAP1, HLA-B, HLA-DOB | 5 | 6.849315 | 5.72E-06 | 1.40E-05 |
| hsa04917:Prolactin signaling pathway | SOCS3, SOCS1, IRF1, CISH | 4 | 5.479452 | 3.14E-05 | 7.31E-05 |
| hsa04660:T cell receptor signaling pathway | CSF2, TNF, NFKBIA, LCP2 | 4 | 5.479452 | 3.05E-04 | 6.60E-04 |
| hsa04151:PI3K-Akt signaling pathway | IL6, IFNA1, IFNB1, CSF1 | 4 | 5.479452 | 6.64E-04 | 0.0013715 |

**Table S3.** Common and unique DEGs in cells infected with wild type and mutant viruses

| **vKO1/vKO2** | **vKO1/vKO2** | **WT virus/vKO1/vKO2** | **WT virus** | **vKO1** | **vKO2** |
| --- | --- | --- | --- | --- | --- |
| BST2 | IL1A | C9 | CDH5 | CASP8 | MR1 |
| C4A/B | IL1RL2 | CCL2 | LILRA1 | CD1D | NFKBIZ |
| CASP1 | IL28A | CCL20 |  | CD45R0 | TICAM1 |
| CCL4 | IL28A/B | CSF2 |  | CEACAM1 | IL23A |
| CCL5 | IL29 | IL8 |  | CLEC7A | TGFBR1 |
| CCL7 | IL6 | PRDM1 |  | CTSS |  |
| CCRL1 | IRF1 | RAG1 |  | CX3CL1 |  |
| CD274 | IRF7 | TNFAIP3 |  | EGR2 |  |
| CD34 | IRF8 | TRAF1 |  | FADD |  |
| CISH | LCP2 |  |  | FAS |  |
| CSF1 | MYD88 |  |  | HLA-DQA1 |  |
| CXCL10 | NFIL3 |  |  | IL18RAP |  |
| CXCL11 | NFKBIA |  |  | IL19 |  |
| CXCL2 | NOD2 |  |  | IL1B |  |
| CXCL9 | PLAUR |  |  | IRF4 |  |
| CXCR4 | PML |  |  | LIF |  |
| EGR1 | PSMB10 |  |  | LY96 |  |
| GBP1 | PSMB8 |  |  | MRC1 |  |
| HLA-B | PSMB9 |  |  | MX1 |  |
| HLA-DOB | RARRES3 |  |  | NOS2 |  |
| ICAM1 | SELE |  |  | TLR2 |  |
| IDO1 | SOCS1 |  |  | TNFSF8 |  |
| IFI16 | SOCS3 |  |  | CTLA4_all |  |
| IFI35 | STAT2 |  |  | ITLN1 |  |
| IFIH1 | TAP1 |  |  | ZAP70 |  |
| IFIT2 | TAP2 |  |  |  |  |
| IFITM1 | TLR3 |  |  |  |  |
| IFNA1/13 | TNF |  |  |  |  |
| IFNB1 | TNFAIP6 |  |  |  |  |
| IL12A | TNFSF10 |  |  |  |  |
| IL15 | TNFSF13B |  |  |  |  |
| IL17F |  |  |  |  |  |

**Supplemental figure legend**

**Figure S1.** **Expression of nsp2-related proteins in HEK-293T cell**. HEK-293T cells seeded in a 24-well plate were transfected with a plasmid (0.5 μg) expressing each of nsp2-related protein. At 24 hpi, immunofluorescence assay was performed to evaluate protein expression. The α-FLAG mAb was used to detect the expression of FLAG-tagged proteins. Rabbit pAb (α-TF) specifically recognizing the unique C-terminal domain of nsp2TF was used to detect the expression of full-length nsp2TF ([Li et al., 2014](#_ENREF_24)), while rabbit pAb (α-nsp2) specifically recognizing the nsp2 C-terminus was used to detect the expression of full-length nsp2 ([Guo et al., 2016](#_ENREF_12)). Cell nucleus was stained with DAPI.

**Figure S2.** **Effect of nsp2-related proteins on HEK-293T cell viability.** Cell viability was determined using a CellTiter 96 AQ_ueous_ cell proliferation assay (Promega). Viability of transfected cells were compared to that of untreated control cells (100%). The average and standard deviation (SD) of a representative experiment are shown. All experiments were repeated twice, and triplicates were performed at each time.

**Figure S3. Protein-protein interaction networks of DEGs in cells infected with WT virus and nsp2TF/nsp2N-deficient mutants.** Protein-protein interaction networks were constructed for all DEGs in cells infected with WT virus (A-B), vKO1 (C-H), and vKO2 (I-N) using the search tool for the retrieval of interacting genes/proteins (STRING). Confidence view of protein-protein interaction network from DEGs showing the strength of data support is indicated by the thickness of the grey line connecting genes and nodes. The representative pathways enriched in cells infected with WT virus (A-B), vKO1 (C-H), and vKO2 (I-N) were highlighted with red color.
